# First-hand experiences of belonging among child refugees and asylum seekers, post-migration: a meta-synthesis

**DOI:** 10.3389/fpsyg.2025.1603733

**Published:** 2025-10-22

**Authors:** Emily F. Trotter, Kate A. Woodcock

**Affiliations:** School of Psychology, University of Birmingham, Birmingham, United Kingdom

**Keywords:** belonging, post-migration, child refugees, unaccompanied asylum seeking children, meta-synthesis, qualitative

## Abstract

**Introduction:**

Refugees and asylum seekers represent a growing population worldwide, of which almost half are children. Child refugees are especially vulnerable and marginalised, yet research into refugee experiences infrequently captures their voices. Establishing a sense of belonging is a particularly pertinent issue for young refugees; however, the processes supporting belonging are poorly understood. This meta-synthesis collated the findings of qualitative studies that explored first-hand experiences of belonging among child refugees and asylum seekers, post-migration, with the aim of better understanding the processes that facilitate a sense of belonging in this population.

**Methods:**

Systematic searches of four electronic databases: PsycINFO, Web of Science, PubMed, and Education Resources Information Centre [ERIC], identified 1,192 primary studies, of which eight were included for meta-synthesis. An integrated qualitative appraisal checklist was used to assess quality of the studies (American Psychological Association, 2018; National Institute of Health and Care Excellence, 2012). The analysis was subsequently guided by Noblit and Hare’s (1988) seven-phase methodology.

**Results:**

One overarching theme was interpreted from the analysis: Migratory Loneliness and Societal Isolation. This underpinned three themes which described the processes facilitating a sense of belonging: Experiences of Inclusion and Support, Family Connectedness, and Adaptive Responses to Resettlement.

**Discussion:**

Findings are discussed in relation to existing research, and clinical implications considered. The study offers insights into the nuances of refugee children’s lived experiences, alongside recommendations for the construction of safe and inclusive spaces where children feel visible. An individualised approach to working with newly resettled children is also advocated.

## 1 Introduction

Over the past several decades, migration has become a growing global phenomenon, whereby migrant families have left, or been forced to leave, their home countries to seek safety and refuge (Pieloch et al., 2016; Rodriguez and Dobler, 2021). The impact of this experience on people’s health and wellbeing is well documented in the literature, as an unprecedented number of people have had to leave behind the land and communities they know, to resettle in unfamiliar countries (Hirad et al., 2022; Radjack et al., 2021).

By the end of 2021, 89.3 million people worldwide were forcibly displaced (United Nations High Commissioner for Refugees, 2022a); that is, involuntarily fleeing their country of origin as a result of economic, social and political upheaval (Salami et al., 2021). For many, this has meant having to escape persecution, conflict, violence, poverty, natural disasters, human rights violations and/or events seriously disturbing public order (Harunogullari and Polat, 2017; Kumi-Yeboah et al., 2020; United Nations High Commissioner for Refugees, 2022a). Since this figure was published, the war in Ukraine has led to an updated figure of more than 100 million displaced people for the first time on record, the largest humanitarian crisis since the Second World War (Ioffe et al., 2022; United Nations High Commissioner for Refugees, 2022b). Of the total forcibly displaced population, 36.5 million (41%) are children below the age of 18 (United Nations High Commissioner for Refugees, 2022a), and a significant proportion are unaccompanied asylum seeking children (UASC). Since 2010, the number of UASC is reported to have increased fivefold in more than 80 countries around the world (Alarcón et al., 2021).

### 1.1 Refugee and asylum seeking children: the challenges they face

There is a great deal of heterogeneity in the refugee community (Crawford, 2016; Hastings, 2012); however, there are numerous daunting challenges that youth from refugee backgrounds are likely to share (Jensen et al., 2014; Lee et al., 2012; McDiarmid et al., 2021). For example, many will have separated from or lost family members, friends and possessions in the process of escaping threatening situations in their country of origin, which may have included destruction of their homes, physical and sexual abuse, and/or deprivation of their human rights (Hastings, 2012). Further, after what can often be a life-threatening escape journey, it is not uncommon for young people to have to deal with uncertainty regarding their legal rights, isolation, exploitation and discrimination, adjusting to which can lead to a range of health difficulties, notably anxiety and depression (McCormack and Tapp, 2019; Sime and Fox, 2015). These difficulties may be compounded by threats to their sense of safety and security, including well-founded fears of discovery and imprisonment, being placed in inadequate housing and/or with emotionally detached caregivers (Hastings, 2012; Weine et al., 2014). Meanwhile, many face the challenge of having to settle into a new educational environment, whilst trying to learn the language and culture of their new host country (Crawford, 2016). Given these challenges, it is perhaps unsurprising that youth from refugee backgrounds consistently show higher levels of psychological morbidity when compared to children from non-refugee backgrounds (Horswood et al., 2019; Reinelt et al., 2016; Van Os et al., 2020).

For many young refugees, the experience of displacement and resettlement coincides with a significant developmental life stage, thereby interrupting the normative processes and transitions that adolescents typically go through (Mayoma and Williams, 2021; Pieloch et al., 2016). Although all adolescents can experience difficulties with ‘fitting in’, refugee adolescents are unique insofar as having been forced to leave their homes and seek asylum elsewhere, and thus face specific challenges to their evolving identities and their experience of belonging (Jardim and da Silva, 2021; Kia-Keating and Ellis, 2007; Meloni, 2019; Miller et al., 2018).

### 1.2 What does it mean to belong?

A sense of belonging is a fundamental aspect of psychological functioning, and a universal human need (Baumeister and Leary, 1995). In social psychology, the need to belong is described as an intrinsic motivation to form connections with others and be socially accepted (Cherry, 2021). Moreover, Amina et al. (2021) referred to belonging as a meaningful and transformative experience; one that helps people to develop emotional connections with their physical and emotional world, laying the foundations to their identity. Notwithstanding these descriptions, belonging is a complex concept and difficult to define. With no uniform definition, it is acknowledged in the literature as a multifaceted, nonlinear and subjective experience; one which is understood from different positions, cultures and contexts (Allsop and Chase, 2017; Lähdesmäki et al., 2016).

From the position and context of the young refugee who has left life as they know it behind, the meaning of belonging is particularly acute (Malsbary, 2013); a notion that is aptly captured by Nunn et al. (2022, p. 43), who described belonging as a “frequent casualty of forced migration.” In the extant literature concerning this population, belonging has been discussed in terms of establishing a ‘sense of place’; that is, the opposite of being displaced (Anthias, 2006; Jardim and da Silva, 2021). This highlights the way in which studies seem to be shifting beyond the individualistic focus typically found in youth policy and practise (Moensted, 2020), to considering the impact of social and systemic change on young people. Such a shift has brought people’s lived experiences into better focus, including their interconnectedness and participation within communities. A sense of belonging is a particularly pertinent issue for adolescent refugees, as the support they receive post-migration is likely to affect how they manage the combined transitions of adolescence and resettlement (de Anstiss et al., 2019). Furthermore, a number of researchers have reported that, for school-aged children in particular, the processes supporting belonging, whilst attempting to negotiate different cultural beliefs, are poorly understood (Green and Thorogood, 2018).

### 1.3 Understanding child refugee perspectives

Given the lack of success in prior attempts to support belonging in young refugees, there is clear value in unpacking their lived experiences and understanding how they establish a sense of belonging during resettlement. However, the existing literature tends to be from the perspectives of parents, teachers or clinicians working with or observing child refugees (Amitay, 2021; Arar et al., 2019; Barber, 2021; Basaran, 2020; Ceballo-Vacas and Trujillo-González, 2021; De Graeve, 2017; Fichtner and Trần, 2020; Kardeş and Kozikoğlu, 2021; Renzaho et al., 2017; Wille et al., 2019). Whilst some studies have attempted to capture the voices of young refugees themselves, the majority focus on children with refugee parents, who have not migrated themselves (Bhambra, 2021; Cunningham and King, 2018; Dusi et al., 2015; Dvir et al., 2015; Korpela, 2016; Orupabo et al., 2019; Zayas and Gulbas, 2017), those who are now adults, reflecting on their past experiences of migration (Nunn et al., 2022; Ruth et al., 2019; Scutaru, 2021; Shmulyar Gréen et al., 2021; Sone and Thang, 2020; Uptin, 2021), or those who migrated voluntarily (Auger-Voyer et al., 2014; Marcu, 2012; Sime, 2020). To date, the literature seems to be lacking representation of young refugee or asylum seeker voices currently undergoing, or recently having undergone, the resettlement process.

### 1.4 Rationale and aim

Young refugees and asylum seekers are often represented as the most excluded, stigmatised and disempowered people in the world (Wilding, 2009), yet research infrequently includes first-hand perspectives of this marginalised population. As a result, there is a lack of knowledge of the unique belonging experiences of refugee children (Welply, 2015), or the processes facilitating belonging, post-migration (Woodgate and Busolo, 2018).

The limited studies on refugee youth tend to be quantitative and expert-driven, with less attention given to the meaning attached to experiences (Edge et al., 2014). Given that child refugees are a hard-to-reach population (Majumder, 2019), any efforts to capture their voices would offer fruitful implications for research and practise, through a better understanding of how to support their integration. The paucity of qualitative research into first-hand refugee experiences indicates a topical and timely area in need of greater research attention. Furthermore, the concept of belonging has become an increasingly relevant social phenomena within both migration and youth studies (Jardim and da Silva, 2021). Therefore, this study aims to identify and evaluate studies that explicate the voices of child refugees and asylum seekers, including UASC. Guided by the theoretical perspective that belonging is a multidimensional and socially constructed experience, the study sought to answer the following research question:

*What are the processes facilitating a sense of belonging in young refugees and asylum seekers during their resettlement?*

### 1.5 A note on terms used

Before proceeding further, it is worth noting that the term ‘refugee’^1^ is highly disputed (Crawford, 2016) and thus considered in further detail and context in Supplementary material A.

## 2 Methodology

The methodological protocol was developed in accordance with the preferred reporting items for systematic review and meta-analysis protocols (PRISMA-P) guidelines (Moher et al., 2015). The literature search strategy was informed by the mnemonic, PICOS^2^ (Richardson et al., 1995; Moher et al., 2009), which is outlined in Supplementary material B.

Given that the research aims to explore lived experiences, a qualitative study design was selected. Qualitative research typically facilitates an exploratory approach into how people interact with and interpret their world (Atkins et al., 2008; Malterud, 2001), otherwise known as a double hermeneutic (Giddens, 1984). Individual qualitative studies can provide rich insights into a specific population of interest. However, the approach has attracted criticism over the years for merely summarising stand-alone findings and not drawing connections between studies (Nye et al., 2016), thus limiting their usefulness in understanding a population or phenomenon.

Qualitative synthesis approaches go beyond summarising findings, by systematically reviewing, interpreting and merging evidence across studies (Lewin et al., 2018). In so doing, another level^3^ of findings is generated through the advancement of theory and knowledge (Nye et al., 2016). After consulting Kastner et al.’s (2016) conceptual recommendations^4^ (Supplementary material C), a meta-synthesis was considered the most applicable method for answering the research question, and enriching understanding of complex experiences and contexts. This was informed by Noblit and Hare’s (1988) seven-phase methodology (Table 1), which serves as a comprehensive guide to the comparative analysis and synthesis of interpretive studies, otherwise known as meta-ethnography^5^.

**Table 1:** Noblit and Hare’s (1988) seven-phase methodology.

Phases	How phases were actioned
1. Getting started	Deciding the focus of the synthesis: Reading literature to develop interest and understanding of chosen area, developing research question according to PICOS.
2. Deciding what is relevant	Preliminary scoping: Developing the search strategy, discussions with research support team, conducting the main search, full text screenings, study selection and quality appraisal.
3. Reading the studies	Repeated reading of studies: Data extraction (study and participant characteristics), paying attention to details (e.g., concepts, themes).
4. Determining how the studies are related	Juxtaposing concepts (metaphors, ideas, phrases) from studies to see how they relate to each other: data extraction (themes), and reducing themes and descriptions to key concepts.
5. Translating the studies into one another	Comparing concepts/metaphors between and within studies.
6. Synthesising the translations	Establishing if there are common types of translations, or if some translations or concepts can encompass those from other studies.
7. Expressing the synthesis	Writing down/articulating themes.

### 2.1 Epistemological position

In conducting this meta-synthesis, a critical realist position was assumed, drawing from a realist ontology and a relativist epistemology. This position acknowledges that there is not one universal truth to be found, but multiple truths (Bhaskar, 1975). In other words, not all child refugees will describe their experiences in the same way. Further, a critical realist stance considers that, whilst participants can provide important information about a phenomenon, it is being understood through a subjective lens and within a socially constructed world (Coyle, 2016).

### 2.2 Search strategy

Preparing the systematic search strategy reflected phases 1–2 of Noblit and Hare’s (1988) methodology, beginning with the iterative process of preliminary scoping of the existing review literature. This guided the subsequent processes of developing inclusion and exclusion criteria, generating search terms, systematic screening, and study selection.

The search strategy was developed following six, monthly workshops, which offered guidance on the different approaches to meta-ethnography, and a space for consultation with other qualitative researchers. This enabled the development of a research support team based at the same university.

#### 2.2.1 Inclusion and exclusion criteria

The population of interest comprised child refugees, including UASC, resettled in any host country that is not their country of origin. In accordance with the United Nations High Commissioner for Refugees (2022b), child refugees and asylum seekers are defined as children, aged 18 years old and under, who have migrated to other countries due to fear of persecution in their home country due to factors such as race, religion, or political opinion. Studies that focus on those who fled their country of origin voluntarily, or internally displaced populations, were not considered for inclusion.

Quantitative studies were not considered for synthesis, but mixed methods were on the condition that the study featured at least one qualitative research question that related to the current study’s aims. Moreover, as this study sought to explore the broad experience of belonging in response to migration and resettlement processes, studies in which the main aim was to evaluate outcomes of an intervention (e.g., family interventions for refugees), were excluded. In terms of the type of publication considered, preliminary scoping indicated that sufficient peer-reviewed journal articles would be available on the topic area to provide data with high information power for addressing the research questions, that dissertations, book chapters, conference publications and grey literature need not be considered.

Given the international nature of refugee research, the researcher considered studies of all languages in the first instance; however, following preliminary scoping, it was decided that papers would be limited to English language. This was due to the researcher’s first language being English and using other languages may result in losing the content and meanings inherent within the original language, once translated. Furthermore, the scoping process indicated that most of the available literature on refugees was published within the last 5–10 years, likely reflecting the growing refugee crisis during this time frame. Studies were thus limited to those that had been published in the last 10 years. Full inclusion and exclusion criteria are summarised in Table 2.

**Table 2:** Inclusion and exclusion criteria.

Inclusion criteria	Exclusion criteria
First-hand accounts given by children (<18 years old at the time of study participation) who have been forcibly displaced and fled their country of origin due to war/conflict, about their recent migration and resettlement experiences.	Studies that focus on the child’s experience from another’s perspective (e.g., parents, teachers, clinicians). Studies in which “child” participant ages range from <18 to >18 (e.g., 16–25), or where age of participants is unclear, or not specified. Studies that focus on retrospective accounts from adults reflecting on their childhood experiences, or children of refugees who have no experience of resettlement themselves. Papers that focus on those who have resettled voluntarily, and/or or internally (within their home country).
Studies reporting original data, published in English and within peer-reviewed journals.	Papers published in a language other than English. Dissertations, books, book chapters/reviews, grey literature (e.g., meeting/conference papers, repositories, white papers, government documents).
Accounts that have been analysed using formal qualitative methods that explore experiences (e.g., thematic analysis [TA], interpretive phenomenological analysis (IPA), case studies, narrative analysis or grounded theory).	Papers using qualitative methods that: Focus on language as opposed to experience (e.g., frequency analysis, discourse analysis. Content analysis), or do not specify what method of qualitative analysis was used or appeared not to use any formal method of analysis.

#### 2.2.2 Developing search terms

As belonging is a fluid concept, and there are many terms to describe refugee children, a range of free-text terms that attempt to operationalise these concepts were compiled by the researcher. These were purposefully simple and broad, to capture a range of potentially relevant studies. The respective search strings were developed after conducting simple Google Scholar searches on the topic areas, making a note of the wording used, and checking synonyms. Preliminary scoping enabled the researcher to filter search terms, discover new terms, and remove those that were found to yield too many irrelevant papers^6^. Based on the research question and scoping outcomes, the terms shown in Table 3 were developed.

**Table 3:** Search terms.

Search string 1 (OR)	AND	Search string 2 (OR)	AND	Search string 3 (OR)	AND	Search string 4 (OR)
Belonging*		Refuge*		Child*		Qualitative
Connectedness		“Asylum seek*”		Adolescen*		Interview*
“Feelings of connection”		“Unaccompanied asylum-seeking child*”		“Young person”		Focus group*
“Perceived connection”		“Undocumented child*”		“Young people”		Ethnograph*
“Fitting in”		Migrant*		Youth		
“Feeling included”		Immigrant*		Teenage*		
“Feelings of inclusion”		“Displaced person”				
“Social inclusion”		“Displaced people”				
Closeness						
“Feeling integrated”						
“Perceived integration”						
“Feeling accepted”						
“Feelings of acceptance”						
“Perceived acceptance”						
“Feeling respected”						
“Feelings of respect”						
“Perceived respect”						
Togetherness						
“Feeling valued”						
“Feeling cared about”						
“Feeling cared for”						
“Sense of community”						
Relatedness						

#### 2.2.3 Information sources

Databases were searched using date restrictions (2012–present), and searched for titles, abstracts and key words, concepts, or topics. ‘English language’ limits were applied, but the option to limit to peer-reviewed articles was only available on two of the databases; therefore, this was determined through the screening process.

Studies were identified after entering the search terms on the following electronic databases. These were systematically searched on 16th January 2022:

- PsycINFO.
- Web of Science (all databases).
- PubMed (National Library of Medicine [NLM]).
- Education Resources Information Centre (ERIC, via EBSCOhost).

The most suitable combination of databases for performing systematic searches remains open to debate. However, it is widely recommended that searches include a combination of broad and subject-specific databases, for optimum coverage and retrieval of the maximum number of relevant references (Bramer et al., 2017).

#### 2.2.4 Data management

Once the search had been performed, all references were exported to Endnote 20, an online reference management software package. Once Endnote had created a complete bibliography of results, the screening process commenced, beginning with the electronic removal of duplicated papers.

### 2.3 Systematic screening process

**Figure 1:**
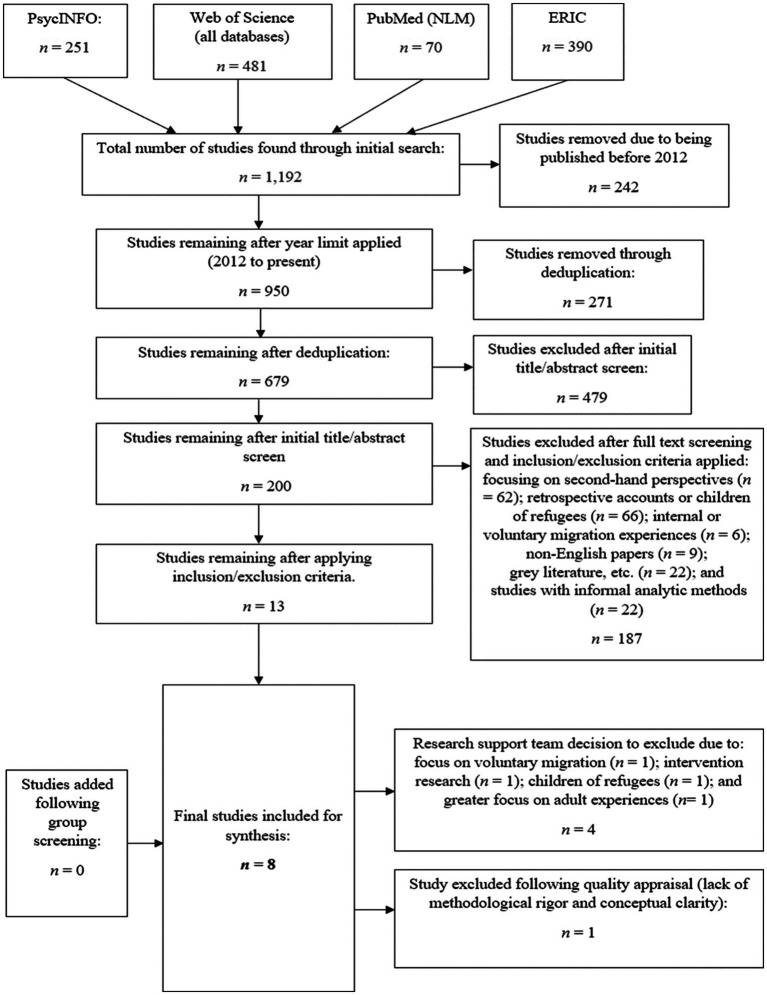
PRISMA-P diagram of the systematic screening process.

Figure 1 illustrates each stage of the screening process, including how and at which point inclusion and exclusion criteria were applied.

Initially, 1,192 potentially relevant studies were identified. Once records had been limited to the last 10 years^7^ and all duplicates removed, the remaining studies underwent title and abstract level screening to remove any unsuitable papers. For the papers with insufficient details in the abstract, a full text screen was completed, and studies that did not meet inclusion criteria were removed. Any duplicates that had evaded the initial deduplication process were also deleted. Next, the remaining studies were subjected to full text screening against the inclusion and exclusion criteria.

Concurrently, three research interns carried out respective title screens, before coming together to cheque levels of inter-rater consensus. Their individual decisions and agreements were documented on a spreadsheet and rated using a ‘traffic light’ system, before consulting the researcher with their justifications. A meeting was then held between the researcher and research interns to discuss the remaining 13 papers, namely those that had not received unanimous agreement. Another full text screen resulted in the exclusion of four papers. A screen capture to illustrate the screening process is shown in Supplementary material D. A total of nine studies remained, all of which were subjected to quality appraisal and data extraction. As shown in the PRISMA-P diagram (Figure 1), one more paper was excluded following the quality appraisal stage, resulting in eight final papers.

### 2.4 Quality appraisal

Reflecting the diversity of qualitative approaches, there is no consensus regarding the necessity, methods or standards for quality appraisal (Majid and Vanstone, 2018). However, to facilitate transferability of findings, appraisal checklists are commonly used (Lewin et al., 2015). For this meta-synthesis, an 18-item appraisal checklist developed by the research support team was used. This integrated two existing checklists: the American Psychological Association (2018) Journal Article Reporting Standards for Qualitative Research (JARS-Qual) and National Institute of Health and Care Excellence (2012) guidance. This contained questions about methodological rigour, transparency, whether researcher bias was considered, and whether ethical procedures were adequately reported, as examples. The researcher added two items regarding whether the studies offered definitions of the key concepts: namely, belonging and child refugees (Supplementary material E, Supplementary Figure E1).

**Figure 2:**
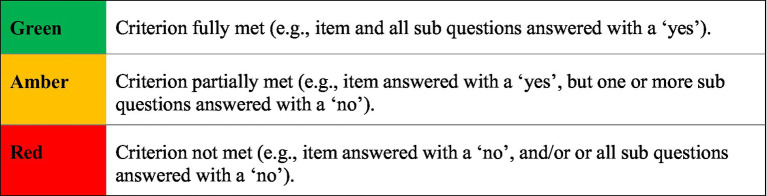
Quality appraisal rating key.

The first stage of quality appraisal involved gathering descriptive data from the final studies in response to the checklist. Then, each study was rated against each checklist criterion using a ‘traffic light’ scoring system, whereby the colours ‘green’, ‘amber’ and ‘red’ denoted whether a criterion was fully, partially, or not met (Figure 2).

**Figure 3:**
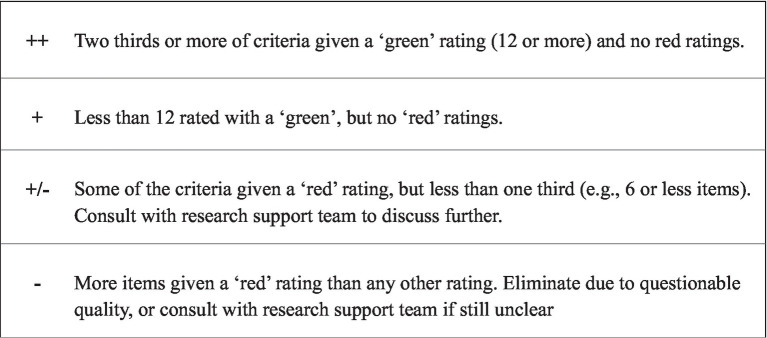
Quality appraisal scoring parameters.

**Figure 4:**
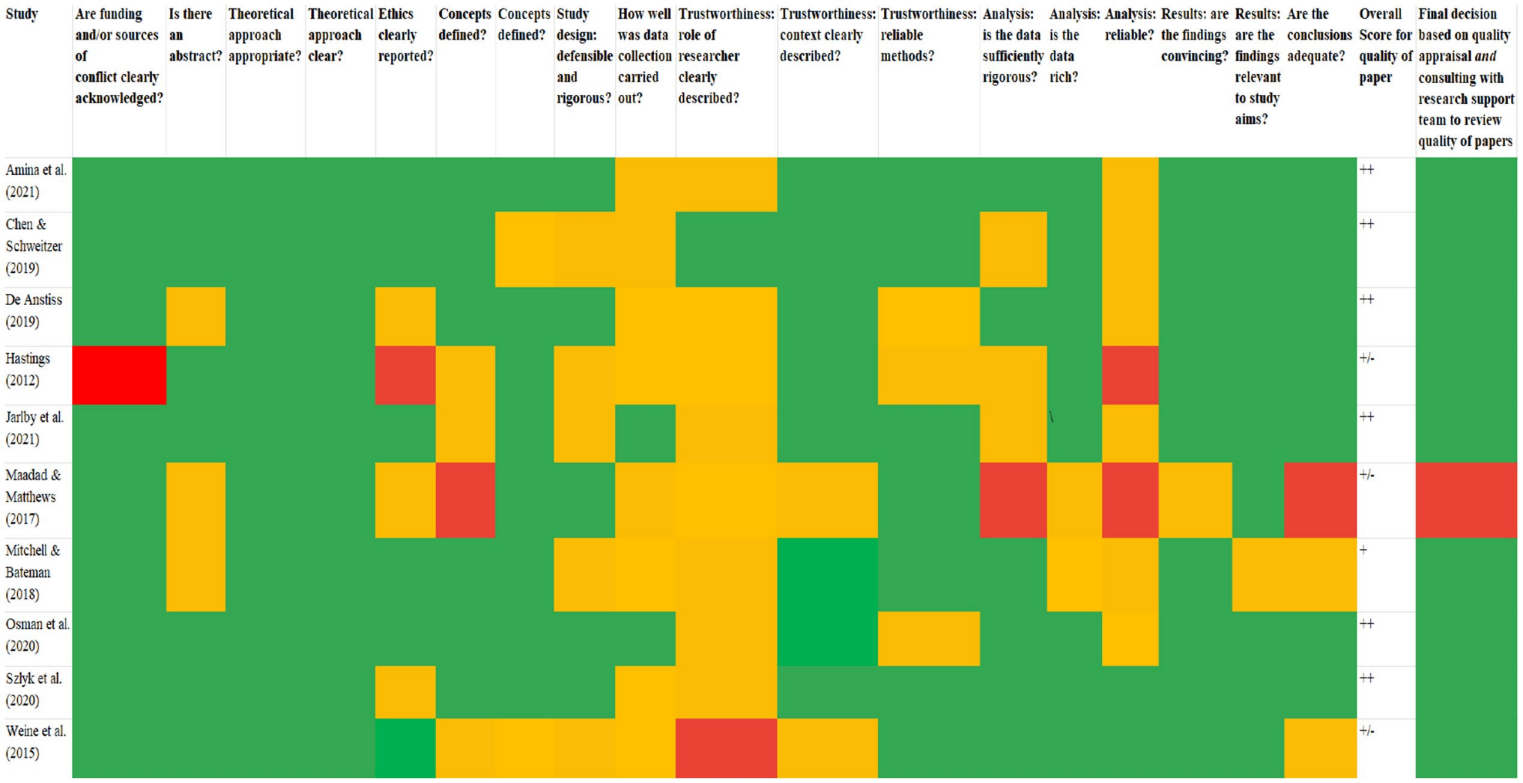
Quality appraisal of studies.

Once all papers had been rated, an overall score for quality was given (Figure 3). This also determined whether any additional studies would be excluded from the meta-synthesis. As depicted in Figure 3, any paper that received a ‘red’ rating was taken to the research support team to discuss any aspects that the researcher felt compromised the overall methodological quality. Then, a collaborative decision was made as to whether the paper would be included or excluded. The outcome of the quality appraisal process, including the final decisions that were reached following consultation with the research support team, are displayed in Figure 4. This process required repeated reading of the final papers, thus reflecting phase three of Noblit and Hare’s (1988) methodology.

Overall, green ratings were assigned more than any other rating. This indicates that the majority of studies met or partially met the full criteria, with common strengths in theoretical approaches; that is, clearly stating study aims, and justifying their decision to use a qualitative approach. The remaining criteria received varied ratings, though quality appeared to consistently fall short when it came to trustworthiness, particularly in relation to describing the role of the researcher(s) and how well data collection was carried out. Although three of the papers received at least one ‘red’ rating (Hastings, 2012; Maadad and Matthews, 2017; Weine et al., 2014), only one stood out as being of questionable methodological quality (Maadad and Matthews, 2017). Two of these papers were discussed for issues such as not clearly reporting ethical procedures (Hastings, 2012), and not clearly describing the role of the researchers (Hastings, 2012; Weine et al., 2014); however, they scored highly in all other areas. Maadad and Matthews (2017) study was the only paper considered to carry a real risk of methodological bias. Specifically, the data analysis was not considered sufficiently rigorous or reliable, and the conclusions did not seem adequate. The paper also lacked conceptual clarity insofar that no attempts were made to define ‘belonging’. For these reasons, the paper was not included for meta-synthesis. Therefore, the quality appraisal process resulted in a total of eight final papers for synthesis.

### 2.5 Data extraction: study and participant characteristics

Data relating to study and population characteristics were extracted from each study, as summarised in Tables 4, 5, respectively.

**Table 4:** Study characteristics.

Author (year)	Country of origin	Host country	Epistemological position	Sample size	Method and analytic tool
Amina et al. (2021)	Afghanistan	Australia	Not stated	*n =* 5	Qualitative: Thematic analysis (TA) and experience drawings
Chen and Schweitzer (2019)	Albania, Afghan, Myanmar, China, Congo, Iran, Eritrea, Ethiopia, Indonesia, Iraq, Samoa, Somalia, Sudan, Syria, Thailand, and Vietnam	Australia	Social constructivist	*n =* 30	Qualitative: Thematic narrative analysis
de Anstiss et al. (2019)	Bosnia, Iraq, Liberia, Iran, Sudan	Australia	Not stated	*n =* 85	Qualitative: TA
Hastings (2012)	Afghanistan, Somalia and Turkey	UK	Not stated	*n =* 6	Qualitative: Interpretative phenomenological analysis (IPA)
Jarlby et al. (2021)	Middle East and South Asia	Denmark	Not stated	*n =* 6	Qualitative: TA and field observations
Mitchell and Bateman (2018)	Burma	New Zealand	Not stated	*n =* 2	Qualitative: TA (with conversation analysis approach)
Osman et al. (2020)	Somalia	Sweden	Not stated	*n =* 47	Qualitative: Thematic network analysis
Weine et al. (2014)	Burundi and Liberia	America	Not stated	*n =* 73	Qualitative: Grounded theory analysis

**Table 5:** Participant demographics.

Author (year)	Mean age (and range) in years	Gender	Ethnicity/Culture
Amina et al. (2021)	Mean not stated (9–12)	1 female	Afghanistan (5)
		4 male	
Chen and Schweitzer (2019)	Mean not stated (13–18)	9 female	Albanian, Afghan, Chinese, Congolese, Eritrean, Ethiopian, Indonesian, Iranian, Iraqi, Karen, Samoan, Somali, Sudanese, Syrian, Thai and Vietnamese
		21 male	
de Anstiss et al. (2019)	Mean not stated (13–17)	41 female	Afghan (16), Bosnian or Serbian (10), Iraqi (17)
		44 male	Liberian (15), Iranian (14), and Sudanese (13)
Hastings (2012)	Mean not stated (12–16)	0 female	Afghan (1), Somalian (4), and
		6 male	Turkish (1)
Jarlby et al. (2021)	Mean not stated (17–18)	0 female	Middle Eastern or South Asian
		6 male	
Mitchell and Bateman (2018)	4 (4)	1 female	Burmese
		1 male	
Osman et al. (2020)	16 (14–18)	25 female	Somalian
		22 male	
Weine et al. (2014)	15.3 (range not stated)	37 female	Liberian (37) and Burundian (36)
		36 male	

All studies were published in Western countries, documenting first-hand accounts of children who had migrated from Eastern countries within the last 5 years. Most studies did not report their epistemological position, with the exception of Chen and Schweitzer (2019). All studies employed a qualitative study design with interpretive methods of analysis. Five papers used a triangulation of multimodal data collection methods, to give voice to linguistically diverse, or younger children. Five studies focused on children’s voices exclusively, whilst three featured accounts of child refugees supported by family members, teachers or service providers (Chen and Schweitzer, 2019; Mitchell and Bateman, 2018; Weine et al., 2014).

The population comprised 254 participants, of which 45% were female (*n* = 114) and 55% male (*n* = 140). A mean age could not be calculated as most of the studies (*n* = 7) did not list individual ages, with five studies reporting age range only. Participants ranged in age from 4 to 18 years old. A possible exception to this was Weine et al. (2014), who only reported the mean age of participants (15.3 years). Consequently, the age of their youngest participant(s) and overall age range is unknown.

### 2.6 Data extraction: themes

Next, the themes from each study were extracted and juxtaposed against each other (Table 6).

**Table 6:** Extraction of themes.

Source			Extracted themes		
Amina et al. (2021)	Theme: The importance of peers as social and linguistic brokers	Theme: The role of teachers’ praise and how recognition increases the students’ sense of belonging	Theme: How inclusive school practises and policies improve the students' sense of membership and participation	**–**	**–**
Chen and Schweitzer (2019)	Theme: Connection to a larger entity	Theme: Experience of immersion	Theme: Experience of connection (and disconnection)	Theme: Sense of identity	Theme: Instrumental outcomes
	Subthemes: (1) a deep experience of the present; (2) a spiritual connection`	Subthemes: (1) positive feelings of comfort and happiness; (2) being submersed in memories of the past	Subthemes: (1) connections to significant others; (2) connections through objects; (3) sense of disconnection and yearning	Subthemes: (1) experience of self in time; (2) sense of agency	Subthemes: (1) obtaining help and support; (2) opportunities for leisure, growth and development
de Anstiss et al. (2019)	Theme: Bonding connections	Theme: Bridging connections	Theme: Linking connections	–	–
Hastings (2012)	Theme: Needing and getting help	Theme: Feeling safe and secure	Theme: Adaptation and belonging	-	-
Jarlby et al. (2021)	Theme: Social support	Theme: Normalcy/acceptance	Theme: Loneliness	Theme: Deviation/exclusion	Theme: Activities (bodily, meaningful, shared)
Mitchell and Bateman (2018)	Theme: Greetings	Theme: Positioning the child within the family and wider cultural community	Theme: Reading	Theme: Dancing	-
Osman et al. (2020)	Global theme: Longing for a sense of belonging	Underlying theme (1): Experience of social exclusion	Underlying theme (2): Pathways to social inclusion and acculturation	-	
	Subthemes/organising themes: Experience of social exclusion; Pathways to social inclusion and acculturation.	Perceived discrimination Lack of supportive adults Swedish language proficiency Cultural in-between-ness	Facilitating immersion Collaborative engagement by teachers and parents Coping and resilience		
Weine et al. (2014)	Theme: Protective agents	Theme: Protective resources	Theme: Protective mechanisms	-	-
	Subcategories (and capacities): (1) Youth (friends, peers); (2) Family (parents, older siblings, extended family members); (3) Service providers (schoolteachers, staff, church congregants, resettlement agency workers, activity leaders, volunteers, health and mental health providers)	Subcategories (e.g., family and community capacities): (1) Finances for necessities; (2) English proficiency; (3) Social support networks; (4) Engaged parenting; (5) Family cohesion; (6) Cultural adherence and guidance; (7) Educational support; and (8) Faith and religious involvement.	Subcategories (and competencies/behaviours): (1) Relational (supporting, connecting, belonging); (2) Informational (informing, preparing); and (3) Developmental (defending, promoting, adapting)		

### 2.7 Conducting the meta-synthesis

It is widely reported that phases 4–6 of Noblit and Hare’s (1988) methodology are the most challenging phases to conduct; yet the literature lacks a rigorous description of how these should be approached in practise (Atkins et al., 2008; Cahill et al., 2018; Sattar et al., 2021). For this reason, the researcher consulted recently developed, practical guidance provided by Sattar et al. (2021) and Toye et al. (2014) who clarified and built on the original steps. It is worth noting here that the original phases are intentionally iterative and cannot be reduced to a set of “mechanistic tasks” (Britten et al., 2002, p. 211). Therefore, whilst the researcher attempted to work closely to recent guidance, they accepted that parallel or overlapping steps would be likely.

One extension to Noblit and Hare’s (1988) original methodology was the collaborative construction of concepts and themes. Qualitative interpretation tends to be richer when two or more researchers are involved (France et al., 2014); therefore, these phases were carried out in collaboration with the research support team. The specific steps are outlined in Table 7.

**Table 7:** Collaborative construction of concepts and themes.

Phase	Steps taken	Rationale
4. Determining how themes were related	The researcher reduced all study themes to key concepts (Metaphors, phrases, or meaningful ideas within the data that develop through comparing instances) and organised them into conceptual categories. Categories were then discussed, challenged and developed within the research support team (reviewers 1 and 2).	Key concepts are the raw data of the synthesis, which attempt to explain and not just describe data.
5. Translating studies into one another	Studies were arranged in order of quality appraisal score, from highest to lowest. The main findings of study one were compared (‘translated’) to study two, and the synthesis of these studies translated into study three, and so on. Areas of similarity, divergence, and study context were noted, and whether each study added anything to the knowledge offered by the last. Participant data were reorganised within the newly formed categories and juxtaposed alongside the primary authors’ interpretations, aiding the development of collaborative interpretations (third order constructs).	The order in which studies are compared can strongly influence the synthesis, as earlier studies will affect the subsequent development of ideas and interpretations. The intention of any qualitative synthesis is to retain the rich context of the data.
6. Synthesising the translations	Two team members independently created visual ‘maps’ to summarise key findings, alongside a textual ‘line of argument’ synthesis to draw relationships between findings. The visual ‘maps’ were then merged, to determine the storey being told by the data as a whole. Reciprocal translations developed by the researcher, and then reviewed by two team members.	A line of argument synthesis describes how findings from across studies identify different aspects that can be drawn together into a new interpretation, ‘storyline’ or model, that may have gone undetected within individual studies.

**Figure 5:**
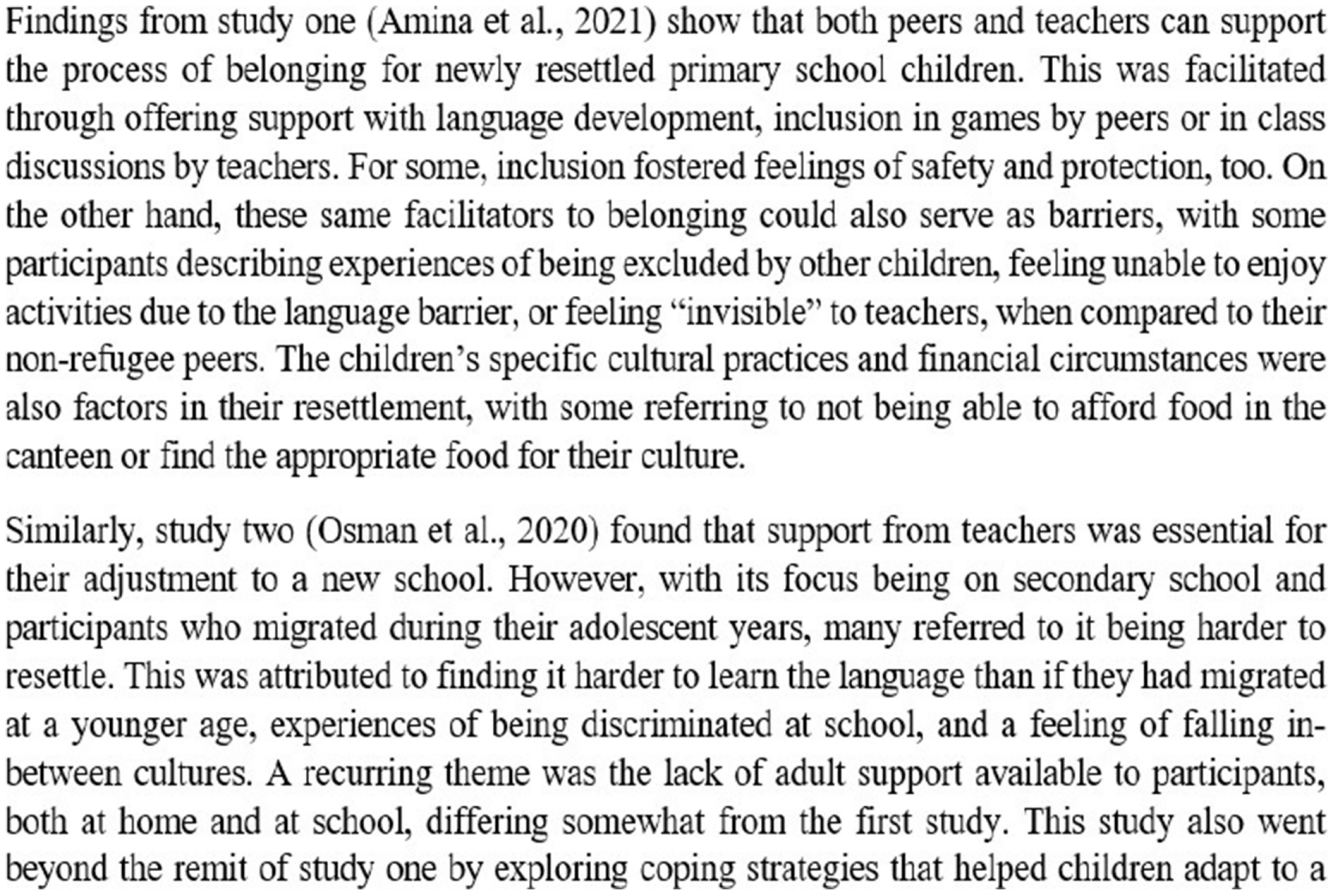
Translating studies into one another.

**Figure 6:**
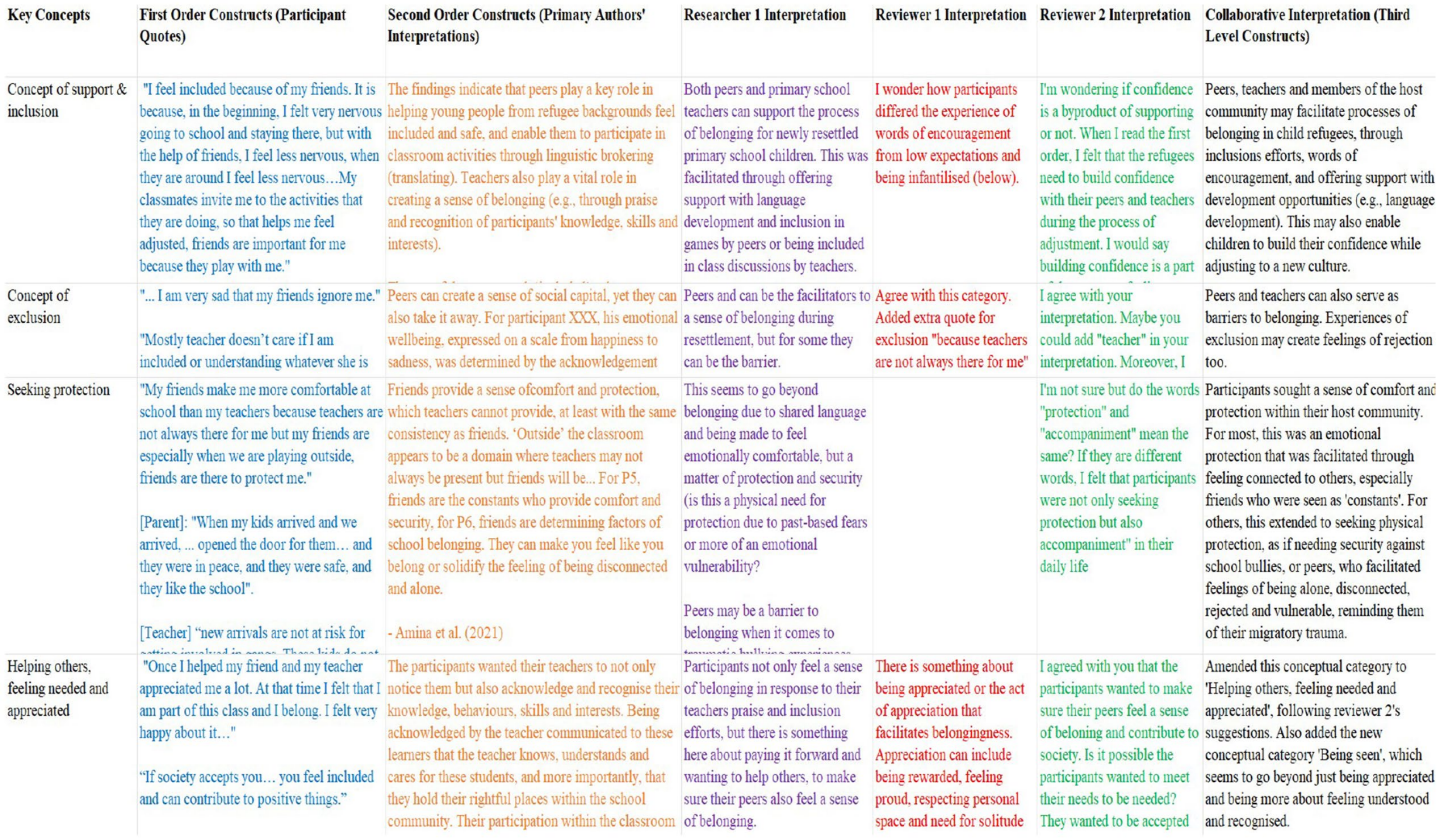
Collaborative translation of studies.

Determining how themes were related resulted in the identification of 25 common concepts (see Supplementary material F). The translating of studies into one another is partially portrayed in Figures 5, 6, and fully in Supplementary material G. The abovementioned steps did not necessitate valuing one team members’ interpretation over another’s; rather, the aim of collaboration was to challenge the researcher’s initial ideas and ensure final interpretations remained grounded in the original studies.

When synthesising the translations, the findings from across the studies were pulled together and viewed as one ‘whole’; one that is more than the parts alone imply (Noblit and Hare, 1988). It had become clear by this phase that the studies were sufficiently similar in focus to warrant a reciprocal synthesis^8^. Practically, this required comparing and merging the translations above into categories of shared meaning (Supplementary material H). This resulted in five reciprocal translations (Table 8). Given the individual differences and circumstances inherent within participant accounts, it was agreed that the available data could not be reformulated into a fixed, linear model of belonging, as the visual ‘maps’ (see Supplementary material I) might have implied. Therefore, the reciprocal translations were collaboratively reviewed and interpreted into new themes, which produced a more narrative description of the processes facilitating a sense of belonging in child refugees.

**Table 8:** Reciprocal translations.

Reciprocal translations	Examples	Number of instances
Experiences of support and inclusion	Feeling included and protected by teachers and peers; feeling seen, needed, appreciated; feelings of family/home; help with language translating.	8
Experiences of exclusion	Feeling ignored, invisible, dehumanised; being attacked and discriminated; language barrier.	8
Migratory loneliness and societal isolation	Like a “boat,” alone at sea; being an ‘outsider’, a ‘foreigner’, a ‘refugee’; stigma and racism; falling between cultures; separated from family.	5
Maintaining a cultural identity	Maintaining connections to country of origin, through language and cultural practises.	7
Adaptive strategies	Embracing opportunities to learn; practising gratitude and self-reliance; engaging in meaningful, shared activities.	7

## 3 Results

The final phase, ‘expressing the synthesis’, marks the reporting of the final themes. One overarching theme was interpreted from the analysis: Migratory Loneliness and Societal Isolation. This represents the wider context in which participants are living, and the context they bring to their resettlement experiences, given their refugee status. This theme underpinned three themes which describe the different types of processes facilitating a sense of belonging, which comprised six subthemes (Table 9).

**Table 9:** Meta-synthesis themes.

Overarching theme	Themes	Subthemes
	Experiences of inclusion and support	Peers as protectors and translators
		Feeling seen, accepted and needed
Migratory Loneliness and Societal Isolation	Family connectedness	A sense of security and cohesion
		Maintaining a cultural identity
	Adaptive responses to resettlement	Embracing agency and new learning opportunities
		Shared and meaningful activities

### 3.1 Migratory loneliness and societal isolation

This theme embodies the overarching lived experience of “being a refugee,” “a foreigner,” and an “outsider.” One participant drew a picture to portray himself as a boat, alone at sea (Jarlby et al., 2021). The ocean represented the world, and he was unsure where he was heading: “Just the world… but it does not mean [that I am] free. It is difficult” (p. 6). Here, this young person seems to be clarifying that whilst he may be physically free now, this does not reflect his felt reality; the fear and loneliness from having to migrate has stayed with him. Later, he explained the meaning of the sun in his drawing: “sometimes the sun is painful, as is your life.” This was echoed in a number of accounts that referred to the sadness and loss experienced through migration.

Many participants alluded to the social impact of migration, especially during the early stages of resettlement (e.g., Hastings, 2012), which placed them in the unique, isolating position of feeling misunderstood from the outset: “I know many people from my school.

But… I cannot really talk with them about my problems… about my life” (Jarlby et al., 2021, p. 186). One participant referred to his worry about being seen as a “freak” from another country (de Anstiss et al., 2019, p. 353), and another expected that societal stigma would accompany their refugee status: “[We will be seen as] extremely scum in their mind… They think, “Oh, [these] people are [all] the same”… that [we] do not want to interact with society, [that we]… want to be isolated…” (de Anstiss et al., 2019, p. 358). These expectations were rooted in perceived experiences of racism and prejudice, which led participants to feeling unwanted in their host community (Jarlby et al., 2021).

Participants’ concerns went beyond their direct experiences, with some referring to how their status dehumanised them from a systemic perspective: “Politicians and the government do not see the human being…” (Jarlby et al., 2021, p. 185). Such concerns appeared to culminate in participants becoming socially withdrawn and generally distrusting of people in their host country, including service organisations (de Anstiss et al., 2019). For these individuals, it was harder to participate within the local community, seek help if needed, and establish a sense of belonging.

Notwithstanding this, whilst many reflected on how their migratory loneliness defined their experiences, one participant reflected on how, with time and increased familiarity with his new environment, things changed:

First day I feel like a lone person, like I do not feel like I belong to this country. When I learn all the laws and about all the people’s behaviours, like I feel now like I belong in this country now (Hastings, 2012, p. 342).

### 3.2 Experiences of inclusion and support

Following migration, a process found to facilitate a sense of belonging was the extent to which participants experienced inclusion and support during resettlement. These experiences were organised into two subthemes: (i) Peers as Protectors and Translators, and (ii) Feeling Seen, Accepted and Needed.

#### 3.2.1 Peers as protectors and translators

Building peer relationships was fundamental for establishing a sense of belonging among child refugees. Participants resettling in primary and secondary school contexts reflected on how they felt nervous and alone at first, but making friends reduced these feelings (Hastings, 2012). Specifically, being included in activities recreated feelings of home, as one participant described: “When I am with my friends, I feel like I’m home… they make me feel better than ever” (Chen and Schweitzer, 2019, p. 1983). This led to feelings of safety and protection in their new environment: “I feel included in my school because of my friends… they help me. My friends make me more comfortable at school than my teachers… friends are there to protect me” (Amina et al., 2021, p. 9).

Furthermore, participants stated their preference for relying on peers for support, because “no one else understands you” (de Anstiss et al., 2019, p. 355). This was especially relevant for those who were able to form attachments with children from their own ethnic or refugee community (Weine et al., 2014), with whom they could share experiences as well as the language, which appeared to instil a sense of camaraderie. Indeed, a factor found to support feelings of inclusion across study contexts, was being able to speak the same language as peers or having peers as linguistic brokers, particularly when they struggled to understand what their teachers were saying (Amina et al., 2021).

These important new relationships in the child’s life, termed ‘protective agents’ by Weine et al. (2014), can facilitate belonging; however, they can also take it away, indicating a continuum of experiences reflecting the extent to which a child is included or excluded.

Some of my friends include me in their games, especially when I am sad… This makes me feel very good… The next one [referring to a picture he has drawn] is about my friends… I am very sad that [they] ignore me (Amina et al., 2021, p. 11).

Many participants described instances of discrimination that were based on being different from their peers. For example, one recalled experiences of being bullied at secondary school due to his inability to speak English: “There were seven boys… they beat me that day” (Hastings, 2012, p. 341). This suggests that, whilst language proficiency can facilitate belonging, the opposite is also true; for those who cannot speak the local language, or do not have peers to translate, language may be a significant barrier (Osman et al., 2020).

#### 3.2.2 Feeling seen, accepted and needed

In a similar vein, for those resettling in school contexts, teachers played a key role in creating a sense of inclusion. Teachers of younger refugee children would encourage belonging by showing how their home culture was valued through using familiar hand gestures and greeting them in their home language (Mitchell and Bateman, 2018). Belonging was also facilitated through observable behaviours reported by older children, such as acknowledging participants in the classroom (Amina et al., 2021), helping with language learning (Chen and Schweitzer, 2019), and generally showing that they cared about the child’s wellbeing and resettlement experiences (Weine et al., 2014). Participants spoke fondly of the teachers who seemed dedicated to supporting newly resettled children and helped them to recognise their future potential (Hastings, 2012). These efforts caused participants to report feeling “important,” “happy” and “confident,” as one participant recalled: “Two months ago, I went to parent/teacher interviews with my mother, my teacher appreciated me… I really felt confident, that made me feel that I really am part of this school” (Amina et al., 2021, p. 11).

Participants also recalled experiences where they had helped others, namely other newcomers to their school, and this had been seen and praised by teachers. For example, one reflected: “Once I helped my friend and my teacher appreciated me a lot. At that time I felt that I am part of this class and I belong. I felt very happy…” (Amina et al., 2021, p. 12). By being appreciated in this way, participants described feeling accepted, needed, and consequently wanting to contribute to their new society through more pro-social acts: “If society accepts you… you feel included and can contribute to positive things” (Osman et al., 2020, p. 8).

As with the previous subtheme, these experiences seemed to occur along a continuum, reflecting the extent to which participants felt included and/or excluded. Some described feeling discouraged, underestimated and ignored by teachers, who were seen as prioritising local children (Amina et al., 2021). This led to children feeling vulnerable, stressed, and hopeless about their future (Osman et al., 2020). Once again, difficulties with language proficiency were identified as a barrier to belonging; participants did not always understand their lessons, nor did they feel cared for or supported to learn, which led one participant to state: “…although I am sitting in front of her, it seems I am invisible. I do not like this” (Amina et al., 2021, p. 12). Moreover, some children described feeling discriminated against by teachers’ comments about their appearance or religion, which seemingly set a precedent for classmates to do the same (Osman et al., 2020).

Across these subthemes, the extent to which participants felt included seemed to be influenced by their: (i) age at migration, and (ii) resettlement context. Those who migrated at a younger age reported fewer transitional difficulties: “I was four or 5 years old when I came to Sweden, so I started preschool… and learned the language more easily” (Osman et al., 2020, p. 7). Older children, however, based in secondary schools, residential settings, the local community and/or service organisations, reportedly found it harder to learn the language and culture, build relationships, and would find themselves drawing comparisons between their own upbringing and current treatment to that of their local peers (de Anstiss et al., 2019).

Moreover, older children seemed more aware of the life they had left behind, and the stigma attached to being a refugee that younger participants did not verbalise (Jarlby et al., 2021).

### 3.3 Family connectedness

A separate process found to facilitate belonging was the extent to which participants felt connected to their family. This process was organised into two subthemes: (i) A Sense of Security and Cohesion, and (ii) Maintaining a Cultural Identity.

#### 3.3.1 A sense of security and cohesion

For those who migrated with family, a sense of security was felt during resettlement, with one participant describing family relationships as “…the most important thing… affecting… every aspect of our life” (de Anstiss et al., 2019, p. 352). This was echoed throughout participant accounts, which referenced the importance of talking through problems they might be having as a family (Weine et al., 2014). Such relationships were thought to protect children against aimlessness, depression and other mental health problems that may arise post-migration (de Anstiss et al., 2019). One participant explained: “…my family stay in my life all my life now and my family lives with me here in Australia, and really help me for everything, and when I’m sad… my family stands up behind me” (Chen and Schweitzer, 2019, p. 1983).

Moreover, a sense of belonging was facilitated when those resettling in school contexts had parents and/or family members that were engaged in this aspect of their life, instilling a sense of cohesion (Weine et al., 2014). In turn, schools that involved the family and values of their home culture when welcoming children were seen as strengthening belonging for younger refugees in particular, as well as helping the family to resettle and embrace new traditions. One parent stated:

We love it, we love the birthdays… we did not have this in our country, it’s not our… way. But … what we see is that our child is alive and for another year. And we are so happy that we want to celebrate that… So that’s what we think now – it’s not just a birthday party… It’s very special (Mitchell and Bateman, 2018, p. 385).

This process seemed to go beyond facilitating a sense of belonging, by helping children and their families to move forward, and in a way that celebrated their survival of the migration experience. In so doing, this parent’s quote implicitly speaks to the life-threatening aspect of being forcibly displaced, and how different things could have been.

#### 3.3.2 Maintaining a cultural identity

Furthermore, proximity to parents enabled participants to maintain their cultural identity. Efforts to remain connected to their country of origin included reminiscing about past memories, the landscape of their home country, sentimental objects, and the important role of family and friends. One participant proudly reflected: “It’s something special about my country. I love my country. I think I’ve learnt [many things in] my country…” (Chen and Schweitzer, 2019, pp. 1982–1983).

For these participants, thinking about their home country seemed to lead them to making sense of how their past and present worlds could integrate coherently to enable a better sense of belonging. On the other hand, sometimes these attempts to remember where they had come from reminded participants of what they had lost, igniting feelings of sadness, yearning and disconnection. Some described feeling like they fell between cultures; something Osman et al. (2020) termed ‘cultural in-betweenness’. For example, one participant explained: “I do not know which country I belong to or which culture I have… I did not grow up in Somalia, and here, I do not celebrate the Swedish midsummer” (Osman et al., 2020, p. 7). Many reported that they strongly identified with their family and home country, but also wanted to engage in the traditions of their new culture (de Anstiss et al., 2019), a desire that was not always supported by parents who were struggling to adjust themselves.

Furthermore, it is important to acknowledge that not all participants felt close to their family, and some were UASC who had been physically separated from family before or during migration. For these participants, it was not uncommon to ruminate about lost connections; however, if they felt included as a member of the local community^9^ and had the chance to talk with others, this seemed to encourage a sense of belonging in the absence of family connections (Jarlby et al., 2021; Weine et al., 2014). For example:

I have many things to think about… why I am alone, where my family is… it could be six or 10 or 20 years that I have to live like this, without a family… I like to talk with people, and if I can help people, it makes me happy (Jarlby et al., 2021, p. 188).

This suggests that ‘family connectedness’ may also sit along a continuum of resettlement experiences which incorporates disconnection too. Many reported low self-esteem because their parents were not involved in their lives (de Anstiss et al., 2019; Jarlby et al., 2021; Osman et al., 2020). For the children in this position, it is inferred that disconnectedness might feed back into feelings of migratory loneliness and impede a sense of belonging.

Some resettlement experiences were also marked by intense conflict and violence at the hands of parents, reportedly due to their own migratory difficulties, which served to maintain the trauma of being forcibly displaced and posed a barrier to belonging:

He (dad) gets abusive… he gets really angry when he drinks… they are always having arguments … And he’s just like “It’s the only way I can like cope with the war and stuff.”… mum’s like, “You cannot always be taking it out on the war.” It’s been like 12 years now and still he does it (de Anstiss et al., 2019, p. 352).

### 3.4 Adaptive responses to resettlement

Across contexts, participants described the processes that helped them to adapt within their new culture. This theme was organised into two subthemes: (i) Embracing Agency and New Learning Opportunities, and (ii) Shared and Meaningful Activities.

#### 3.4.1 Embracing agency and new learning opportunities

Adolescent refugees in particular voiced the bidirectional nature of belonging, with many believing that it was their responsibility to integrate and learn about their new culture, especially for those lacking family connections and other forms of support (Osman et al., 2020). These participants opted to accept and normalise the challenges that come with change and coming to belong in a new country, indicating a sense of agency and emotional maturity beyond their years. One group of participants recalled telling themselves that things will get better with time, with one declaring:

There are challenges and changes in your life, you have to accept these because you have come to a new country, and everything is new… Eventually, you will adapt… you will meet other people who will encourage you (Osman et al., 2020, p. 8).

Many participants embraced opportunities to develop new skills. With this came expressions of gratititude, for their parents, their safety, and the experiences they may not have had if they had remained in their war-torn home country, including free education and healthcare (Chen and Schweitzer, 2019; Osman et al., 2020). A large proportion of secondary school participants valued learning English in particular: “I just want to learn. I just love to learn English” (Hastings, 2012, p. 342). These opportunities allowed participants to nurture hopes and dreams for the future, and think about developing the necessary competencies to create a better life for themselves (Chen and Schweitzer, 2019; Jarlby et al., 2021).

Another way that participants appeared to embrace agency and new learning opportunities was through reflecting on their spiritual beliefs, and the universality of human experience. This implied that belonging may take on a transcendent quality, conceptualised as being connected to something bigger than themselves; a shared earth (Chen and Schweitzer, 2019; Jarlby et al., 2021; Weine et al., 2014). This involved connecting with the present moment, nature, and embracing a sense of communion, regardless of their past and where they have come from, implying a sense of humility: “the word belonging in our language… like, being united, being together, and once people are together… they are one” (Chen and Schweitzer, 2019, p. 1981). Another participant stated:

It does not matter if you are from Africa, Asia, Europe, South America… we are people and we live only in one earth … Being part of a place… It does not matter if you are from outside of Australia… just you are belonging here (Chen and Schweitzer, 2019, p. 1982).

#### 3.4.2 Shared and meaningful activities

Some suggested that a sense of belonging was promoted through membership to recreational teams and opportunities to participate in meaningful activities with others. This allowed participants to nurture hobbies they loved in their home country, or develop new interests altogether, which enabled them to have fun, alleviate migration-related stress, and begin to move forward (Amina et al., 2021; Chen and Schweitzer, 2019; Jarlby et al., 2021; Osman et al., 2020). One participant explained: “Through activities such as sports, you learn how to adapt to society. Playing football with native youths… helps you get to know them and make friends, which… helps you feel happy and able to manage every hardship” (Osman et al., 2020, p. 7).

In addition to the positive experience of the activities in themselves, they were also part of pursuing an ordinary, meaningful life, through which child refugees could access opportunities for bonding and feel a sense of normality. One participant shared: “It makes me happy when I go out with someone, just walking… talking together. Like normal people do…” (Jarlby et al., 2021, p. 188).

As with the preceding themes, divergent experiences were also described. Despite particpants’ willingness to engage in meaningful activitities, not all of them had these opportunities in their life (Jarlby et al., 2021). This was due to the aforementioned challenges of feeling misunderstood, an absence of meaningful relationships, and language barriers.

These were highlighted in accounts of poor health and wellbeing, which led some to report low self-esteem and believe themelves to be “…the most useless thing on the planet” (de Anstiss et al., 2019, p. 354), ultimately obstructing opportunities which may facilitate belonging. Some also resorted to maladaptive ways of manage their pain, from dropping out of school (Osman et al., 2020), to self-harming (de Anstiss et al., 2019; Jarlby et al., 2021).

## 4 Discussion

### 4.1 Summary of findings

The current study aimed to explicate the voices of children from refugee backgrounds, to understand the processes that facilitate a sense of belonging. After systematically searching four electronic databases, 1,192 studies were identified, of which eight primary studies were included for meta-synthesis. Following Noblit and Hare’s (1988) seven-phase methodology, it became clear that there were considerable differences between child refugees’ experiences and personal circumstances within studies. However, the studies were sufficiently similar in focus to warrant a reciprocal synthesis. Subsequently, one overarching theme was interpreted: Migratory Loneliness and Societal Isolation. This underpinned the development of three themes: (i) Experiences of Inclusion and Support, (ii) Family Connectedness, and (iii) Adaptive Responses to Resettlement. These themes comprised a total of six subthemes (see Table 9), each of which offered a narrative description of the processes facilitating a sense of belonging in child refugees.

### 4.2 Connection to existing theory and research

The organising themes are considered in the wider context of the broader literature, noting similarities, differences, and any new ways of understanding the population of interest.

#### 4.2.1 Experiences of inclusion and support

A key process found to facilitate a sense of belonging was building supportive relationships with peers, within school and the local community. Teachers also played a vital role, through accepting newly resettled children and their home cultures. These findings are supported by the wider, recently published literature concerning refugee and immigrant youth, which attests to the importance of developing social support networks post-migration (Garcia and Birman, 2022). Schools have been identified as a particularly important context for facilitating such support, where youth can both learn, and be part of a welcoming environment (Schachner et al., 2016). Corresponding with the current study, Pastoor (2015) found that being able to attend school and build relationships with local peers also promoted stability and a sense of normality during a time that tends to be characterised by coping with traumatic memories and fearing what the future might hold.

Participants’ experiences were interpreted as occurring along a continuum, highlighting how the processes facilitating a sense of belonging for some, may have had the opposite result for others. In keeping with previous research (McCormack and Tapp, 2019; Sime and Fox, 2015), participants across study contexts raised the issue of being excluded and discriminated against. Participants perceived this treatment as being based on difference, from their appearance and how refugees are portrayed by the government, to not being able to speak the local language. Whilst first-hand knowledge of child refugee experiences is lacking in the broader literature, this finding is unsurprising when considering that similar experiences have been reported by young adult refugees (Edge et al., 2014), and children of refugees (Demir and Ozgul, 2019).

The extent to which participants felt included or excluded seemed to be influenced by participants’ age at migration and the context they were resettling in. This finding has been reported in earlier studies which have noted that age of displacement can be a critical factor for refugee children (Rutter, 2003). Moreover, the significance of resettlement context is consistent with Bronfenbrenner’s (1979) ecological systems theory, which supports the idea that the social conditions within a child refugee’s host country, combined with any psychological resources formed in their home country, can affect their wellbeing post-migration. This indicates an additional influencing factor that was not explored within the current study: the impact of pre-migration experiences on belonging.

#### 4.2.2 Family connectedness

Another key process supporting a sense of belonging was the extent to which participants felt connected to their family, which afforded opportunities to remain connected to their country of origin, whilst still feeling supported with integrating into a new community. This too appeared to occur along a continuum, given the stark reality that many participants were UASC and had been separated from their loved ones. In the absence of family connectedness, some participants reported a feeling of falling between cultures, and consequently not belonging to any country.

These findings bear similarities with research carried out with adult individuals and families. For example, Gangamma (2017) found that maintaining family connection during resettlement appeared to help members to make sense of the loss they have experienced. This was largely rooted in their shared trauma experiences and navigating the challenges of resettlement together. However, it is impossible to say whether this interpretation can be extended to reflect child refugees’ experiences.

#### 4.2.3 Adaptive responses to resettlement

Participants described a sense of belonging through embracing opportunities for learning and development, through learning new skills and participating in recreational activities. In recent years there has been a shift within the evidence base, from pathologizing refugee experiences (Garcia and Birman, 2022), to framing their narratives in the context of strengths, resilience and aspirations in resettlement, despite any migration-related stress they may be carrying (Alarcón et al., 2021; Becker et al., 2018; Pieloch et al., 2016; Rodriguez and Dobler, 2021). Reflecting this shift, the current study highlighted narratives in which participants voiced gratitude for their newfound safety, and the new educational opportunities they would not have had if they had remained in their country of origin. The findings therefore contribute to a more positive representation of refugee children in the academic literature, whilst also going some way in dispelling views of vulnerability and resilience as mutually exclusive (Ni Raghallaigh and Gilligan, 2010).

### 4.3 Clinical implications

The current findings are consistent with previous research that has called for an extended scope of treatment for refugees that looks beyond symptoms of distress and trauma and considers the important role of family, peer and community connections when establishing a sense of belonging (e.g., Gangamma, 2017).

At the clinical level, the findings have real world applications for service provision within the local authority sector. For example, some participants described feeling dehumanised by their refugee status, which reportedly made it harder for them to trust service organisations and seek help if needed. This is a particularly worrisome finding given the growing population of child refugees, who bear an increased risk of mental health difficulties as a result of their traumatic experiences prior to, during, or post migration. Adding to this, the current study found that being unable to speak the local language created a barrier to belonging for participants. Although refugees and asylum seekers are entitled to equality of access to mental health services, free emergency care and primary healthcare in the UK (Office for Health Improvement and Disparities, 2017), greater resource support is needed from government (Pollard and Howard, 2021). Moreover, the language barrier represents a significant problem related to accessing these services. Therefore, the current study provides evidence to support the continued commissioning of interpreting services across health services (NHS England, 2018), whilst calling for the recruitment of more culturally and linguistically diverse workforces. At the very least, health information and therapeutic resources should be made available in a range of languages, which would be particularly helpful for those who are distrusting of health care providers, and may struggle with engagement.

Clinical Psychologists could play a vital role in the development of such resources; for example, by developing and disseminating trauma-informed training programmes across contexts, including schools and the care sector. An example of an existing programme that has been rolled out across Europe is Including Children Affected by Migration (2022), which provides resource packs for schools who are welcoming Ukrainian children.

Moreover, a number of child and adolescent services across Europe have opened specialist outpatient units for young refugees and asylum seekers, led by healthcare professionals (Hodes et al., 2018). This may be an additional area requiring psychology input, through providing clinical supervision for the professionals providing direct support, especially given their risk of exposure to vicarious trauma (Barrington and Shakespeare-Finch, 2013). Holding a space for reflective practise may also be beneficial, which could provide time for service providers to think about how to implement culturally appropriate treatment for this vulnerable population (e.g., Pollard and Howard, 2021).

Regarding the educational sector specifically, the findings relating to the support experienced from peers and teachers at school offer important implications. The experience of migratory loneliness is likely to be accentuated by the fact that most refugee children willalso encounter interruptions to their education (Wofford and Tibi, 2018), causing a number of social and learning consequences. This is a timely issue given the recent context of Covid-19 (Browne et al., 2021), which not only brought more interrupted schooling for this population but may have exacerbated the feelings of isolation felt through migration. Access to a supportive educational environment, as a basic human right, should therefore be a priority for newly resettled refugee children. The following recommendations may help to facilitate this process, and thus a sense of belonging.

First, those responsible for child refugee integration^10^ should make efforts to create safe, welcoming environments in which children feel visible and accepted. Reflecting Mitchell and Bateman’s (2018) study, these should include activities that celebrate children’s home cultures, whilst introducing the host country’s own western traditions. Indeed, integration should be a two-way process, whereby participation within a new community also requires encouragement from members of that community (Bouchara, 2021). This seems important given the current findings which suggest that some adolescents can assert agency in their integration efforts; however, this may not be possible for all refugees. For very young refugees, the onus lies with the host society to take a more involved role in accommodating diversity and removing barriers to belonging.

Second, to minimise perceived discrimination, support should be in the form of a holistic, strengths-based approach that builds on refugee children’s goals and previous level of education, not limiting their value to language proficiency. That said, language and literacy development in both the home and host culture languages of refugee children have been found to increase the likelihood of successful adjustment and should therefore be prioritised, at home and in the classroom (Wofford and Tibi, 2018). This reflects recently updated UK policies, including the ‘Home Office indicators of integration framework’ (Ndofor-Tah et al., 2019). The framework identified both social connection and the development of language and cultural knowledge as key facilitators for the positive integration of young refugees in the education sector. With this in mind, when educators within western societies do attempt to teach a language to children from the eastern world, an additional recommendation within the wider literature is that they must also teach them about the corresponding cultural paradigm, including any taboos that may come with it (Bouchara, 2021). Furthermore, to facilitate a sense of cohesion and connection in the child’s new life, educators should attempt to bring parents or caregivers into the conversation when planning language and literacy interventions for their children where possible, or at least offer support to families who may lack the resources to support their children in navigating academic contexts (Zeynep, 2012).

Finally, those supporting children from refugee backgrounds should ensure that all of their actions look past the stigma that can come with ‘being a refugee’, and instead see the human being, and the unique position their experiences have placed them in. As alluded to in previous research, the very definition of being forcibly displaced should also be held in mind when working and communicating with newly resettled children, as a reminder of the non-normative experiences that set them apart from their non-refugee peers (Horswood et al., 2019; Korjonen-Kuusipuro et al., 2019; Van Os et al., 2020). With this in mind, it is felt that a more individualised and trauma-informed approach is warranted with this population; one that recognises a person’s lived experiences and resulting adjustment strategies, whilst respecting their cultural and religious identity (Sobitan, 2022). This was shrewdly captured by a teacher in one of the primary studies:

I wondered how much of [their] behaviour might be survival strategies learned previously, behaviours and mentalities that might have been necessary to survive in conditions of scarcity and uncertainty. Part of addressing their behaviour is to create an environment that is safe and predictable, and where every student is assured of having everything they need in the classroom. Not needing to fight to get what they want (Weine et al., 2014, p. 14).

### 4.4 Future research

The processes described within the first two organising themes were interpreted as existing along a continuum of experiences. However, it was not clear whether any one participant experienced either inclusion or exclusion experiences, for example, or both.

Similarly, it was not known whether participants who felt included by peers and/or teachers, were in a position to maintain family connections or not, reflecting a limitation of the available data and an area worthy of further exploration. Previous research has found that support from peers, but not family, to be a predictor of successful adaptation in UASC (Tartakovsky, 2009), implying that a sense of belonging in one context may counteract a lack of belonging in others. However, these studies were not based on forcibly displaced youth, making it difficult to draw inferences in relation to participants within the current study, indicating a potential area for future research attention. Such research efforts may also capture broader insights into young refugee experiences by exploring their migration and pre-migration experiences, to better contextualise their resettlement experiences and how a sense of belonging is established.

### 4.5 Limitations

Despite this study’s contributions, several limitations were noted. First, the raw data of meta-syntheses are participant narratives selected by the primary studies’ authors to support their interpretations, meaning the current findings are limited to what the original authors chose to present. As the majority of primary studies did not report their epistemological position, potentially valuable information is lost regarding any beliefs or biases of the authors’ that may have influenced how they made sense of participants’ experiences.

Considering the multicultural nature of the studies being reviewed and the range of participant languages, an initial concern of the researcher, as an English-speaking, monolingual female, was that she might have missed important individual differences between participants and their experiences when translating studies into one another.

However, this issue is somewhat mitigated by the collaborative approach to analysis, through which the researchers’ individual interpretations were challenged and developed at each phase. Discussions with the research support team, who represented a diverse population^11^, also involved exploring any *a priori* assumptions or biases held by the researcher, enabling an iterative, dialectic and thus, more rigorous process (Toye et al., 2014).

### 4.6 Strengths

Though concerns have been raised within previous literature about the potential loss of explanatory context when combining the findings of multiple studies (Atkins et al., 2008), the current study countered these by synthesising a small number of studies, as recommended by Campbell et al. (2011)^12^. The final studies also represented a range of contexts, each of which were acknowledged when translating studies into one another. The studies combined also represented voices from a large sample of children (*n* = 254), aged 4–18 years, who had migrated from at least 22 countries in the Eastern world, thus capturing a broad but in-depth range of child refugee experiences.

Another strength was that the analysis explored both complementary and contradictory data, offering a more holistic and contextualised understanding of the range and complexity of resettlement experiences. This is supported by Haarlammert et al. (2017), who contended that representing just one aspect of a storey can inadvertently “other” refugee children. This meant that the study adhered to representational ethics and facilitated a richer synthesis of findings, whilst still remaining close to the research question.

## 5 Conclusion

The current meta-synthesis offered insights into the nuance of child refugee experiences and circumstances, and how these can vary between individuals depending on their age and resettlement context. Perhaps more importantly, the study provided a platform from which child refugee voices could be heard, thus contributing to a significant gap in the literature and paving the way for further developments to clinical practise and research into the processes facilitating a sense of belonging.

## Data Availability

The original contributions presented in the study are included in the article/Supplementary material, further inquiries can be directed to the corresponding author.

## Footnotes

^1^For the purpose of this chapter, the terms ‘young refugee’ and ‘child refugee’ will be used interchangeably and are intended to cover the wide spectrum of displaced youth, including UASC, and those who have migrated with their families.

^2^Population, Intervention or exposure, Comparators, Outcomes, Study design or type (PICOS).

^3^Frequently referred to as ‘higher order’ or ‘third order constructs’ (where first order constructs are the primary data, and second order constructs are the primary authors’ interpretations of that data).

^4^Conceptual algorithm to optimise selection of a knowledge synthesis method for answering a research question.

^5^An interpretative, inductive approach upon which most qualitative synthesis methods are based, particularly suited to the development of conceptual models and theories.

^6^“Unaccompanied asylum-seeking child*,” “undocumented child*” and “displaced person”/“people” were added, and “minor” was removed due to generating more studies of minority groups than young refugees.

^7^10-year time frame was specific to the search date. I.e., a search carried out on 16th January 2022 meant that the date filter would only allow studies published on or after 16th January 2012.

^8^When the concepts in one study can incorporate those of another.

^9^Including neighbours, volunteer agencies, the local church and other children.

^10^Including teachers, clinicians, residential care staff, policymakers, community workers, etc.

^11^Collaborative efforts involved the researcher, a Caucasian female, and two reviewers; one was a north African female, and the other an Asian male. Three Caucasian supervisors were also involved in the process. Though culturally diverse, a caveat may be that the research support team were exclusively formed by clinical psychology trainees or supervisors.

^12^Campbell et al. (2011) recommended <40 papers for an effective meta-synthesis.

## References

[ref1] Alarcón X. Bobowik M. Prieto-Flores Ò. (2021). Mentoring for improving the self-esteem, resilience, and hope of unaccompanied migrant youth in Barcelona metropolitan area. Int. J. Environ. Res. Public Health 18, 1–25. doi: 10.3390/ijerph18105210 PMC8156929 34068880

[ref2] Allsop J. Chase E. (2017). Best interests, durable solutions and belonging: policy discourses shaping the futures of unaccompanied migrant and refugee minors coming of age in Europe. J. Ethn. Migr. Stud. 45, 293–311. doi: 10.1080/1369183X.2017.1404265

[ref3] American Psychological Association (2018). Information recommended for Inclusion in manuscripts that report primary qualitative research. Available online at: https://apastyle.apa.org/jars/qual-table-1.pdf (accessed February 2, 2022).

[ref4] Amina F. Barnes M. M. Saito E. (2021). Belonging in Australian primary schools: how students from refugee backgrounds gain membership. J. Multiling. Multicult. Dev. 43, 1–24. doi: 10.1080/01434632.2022.2026367

[ref5] Amitay G. (2021). From helpless rage to loving resistance: resistance to othering and practices of agency in mentoring children of asylum seekers in Israel. Child Youth Care Forum 51, 705–727. doi: 10.1007/s10566-021-09649-7

[ref6] Anthias F. (2006). “Belongings in a globalising and unequal world: rethinking translocations” in The situated politics of belonging. eds. Yuval-Davis N. Kannabiran K. Vieten U. (SAGE Publications Ltd.), 17–31.

[ref7] Arar K. Örücü D. Küçükçayir G. A. (2019). A holistic look at education of the Syrians under temporary protection in Turkey: policy, leadership and practice. Int. J. Leadersh. Educ. 23, 7–23. doi: 10.1080/13603124.2019.1603401

[ref8] Atkins S. Lewin S. Smith H. Engel M. Fretheim A. Volmink J. (2008). Conducting a meta-ethnography of qualitative literature: lessons learnt. BMC Br Med Res Methodol 8, 1–10. doi: 10.1186/1471-2288-8-21 PMC2374791 18416812

[ref9] Auger-Voyer V. Montero-Sieburth M. Perez L. C. (2014). Chasing the European dream. Unaccompanied African youths’ edicational experience in a Canary Islands’ reception Centre and beyond. Educ. Policy Anal. Arch. 22, 1–23. doi: 10.14507/epaa.v22n76.2014

[ref10] Barber S. (2021). Achieving holistic care for refugees: the experiences of educators and other stakeholders in Surrey and greater Vancouver, Canada. Br. Educ. Res. J. 47, 959–983. doi: 10.1002/berj.3730

[ref11] Barrington A. J. Shakespeare-Finch J. (2013). Working with refugee survivors of torture and trauma: an opportunity for vicarious post-traumatic growth. Couns. Psychol. Q 26, 89–105.

[ref12] Basaran S. D. (2020). Becoming a teacher for Syrian refugee students: teachers’ school experiences. Eğitim ve Bilim 46, 331–354. doi: 10.15390/EB.2020.9182

[ref13] Baumeister R. F. Leary M. R. (1995). The need to belong: desire for interpersonal attachments as a fundamental human motivation. Psychol. Bull. 117, 497–529. doi: 10.1037/0033-2909.117.3.497, PMID: 7777651

[ref14] Becker R. Sabet R. F. Swanson A. Suarez L. G. Marques D. S. Ameen E. J. . (2018). “They were going to kill me”: resilience in unaccompanied immigrant minors. Counsel. Psychol. 46, 241–268. doi: 10.1177/0011000018759769

[ref15] Bhambra M. (2021). Perceptions, experiences and accommodations of Britishness; an exploration of national identity amongst young British Sikhs and Hindus in London. Natl. Ident. 24, 1–21. doi: 10.1080/14608944.2021.1935836

[ref16] Bhaskar R. (1975). A realist theory of science. York: Books.

[ref17] Bouchara A. (2021). Taboos as a cultural cleavage between Muslim immigrants and secular western publics: bridging the gaps by viewing integrations as a two-way process. Islamophobia Stud. J. 6, 228–246. doi: 10.13169/islastudj.6.2.0228

[ref18] Bramer W. M. Rethlefsen M. L. Kleijnen J. Franco O. (2017). Optimal database combinations for literature searches in systematic reviews: a prospective exploratory study. Syst. Rev. 6, 1–12. doi: 10.1186/s13643-017-0644-y 29208034 PMC5718002

[ref19] Britten N. Campbell R. M. Pope C. J. Donovan J. L. Morgan M. Pill R. (2002). Using meta-ethnography to synthesise qualitative research: a worked example. J. Health Serv. Res. Policy 7, 209–215. doi: 10.1258/135581902320432732 12425780

[ref20] Bronfenbrenner U. (1979). The ecology of human development. Cambridge, MA: Harvard University Press.

[ref21] Browne D. T. Smith J. A. Basabose J. D. D. (2021). Refugee children and families during the COVID-19 crisis: a resilience framework for mental health. J. Refugee Stud. 34, 1138–1149. doi: 10.1093/jrs/feaa113

[ref22] Cahill M. Robinson K. Pettigrew J. Galvin R. Stanley M. (2018). Qualitative synthesis: a guide to conducting a meta-ethnography. Br. J. Occup. Ther. 81, 129–137. doi: 10.1177/0308022617745016

[ref23] Campbell R. Pound P. Morgan M. Daker-White G. Britten N. Pill R. . (2011). Evaluating meta-ethnography: systematic analysis and synthesis of qualitative research. Health Technol. Assess. 15, 1–164. doi: 10.3310/hta15430, PMID: 22176717

[ref24] Ceballo-Vacas E. Trujillo-González E. (2021). Emotional difficulties and support of migrant students: a case study in a multicultural secondary school. Open Classroom 50, 767–776. doi: 10.17811/rifie.50.4.2021.767-776

[ref25] Chen S. Schweitzer R. D. (2019). The experience of belonging in youth from refugee backgrounds: a narrative perspective. J. Child Fam. Stud. 28, 1977–1990. doi: 10.1007/s10826-019-01425-5

[ref26] Cherry K. (2021). What is the sense of belonging? Verywell Mind. Available online at: https://www.verywellmind.com/what-is-the-need-to-belong-2795393 (accessed January 28, 2022).

[ref27] Coyle A. (2016). “Introduction to qualitative psychological research” in Analysing qualitative data in psychology. eds. Lyons E. Coyle A. (London: Sage), 9–31.

[ref28] Crawford R. (2016). Creating unity through celebrating diversity: a case study that explores the impact of music education refugee background students. Int. J. Music. Educ. 35, 343–356. doi: 10.1177/0255761416659511

[ref29] Cunningham U. King J. (2018). Language, ethnicity, and belonging for the children of migrants in New Zealand. Int. J. Bilingual. 8, 1–11. doi: 10.1177/2158244018782571

[ref30] de Anstiss H. Savelsberg H. Ziaian T. (2019). Relationships in a new country: a qualitative study of the social connections of refugee youth resettled in South Australia. J. Youth Stud. 22, 346–362. doi: 10.1080/13676261.2018.1508824

[ref31] De Graeve K. (2017). Classed landscapes of care and belonging: guardianships of unaccompanied minors. J. Refug. Stud. 30, 71–88. doi: 10.1093/jrs/fev011

[ref32] Demir S. B. Ozgul V. (2019). Syrian refugees minors in Turkey. Why and how are they discriminated against and ostracised? Child Indic. Res. 12, 1989–2011. doi: 10.1007/s12187-019-9622-3

[ref33] Dusi P. Messetti G. González Falcón I. (2015). Belonging: growing up between two worlds. Procedia. Soc. Behav. Sci. 171, 560–568. doi: 10.1016/j.sbspro.2015.01.161

[ref34] Dvir N. Aloni N. Harari D. (2015). The dialectics of assimilation and multiculturalism: the case of children of refugees and migrant workers in the Bialik-Rogozin school, Tel Aviv. Compare J. Comp. Int. Educ. 45, 568–588. doi: 10.1080/03057925.2014.884335

[ref35] Edge S. Bruce Newbold K. McKeary M. (2014). Exploring socio-cultural factors that mediate, facilitate, and constrain the health and empowerment of refugee youth. Soc. Sci. Med. 117, 34–41. doi: 10.1016/j.socscimed.2014.07.025, PMID: 25036014

[ref36] Fichtner S. Trần H. M. (2020). Lived citizenship between the sandpit and deportation: young children’s spaces for agency, play and belonging in collective accommodation for refugees. Childhood 27, 158–172. doi: 10.1177/0907568219900994

[ref37] France E. F. Ring N. Thomas R. Noyes J. Maxwell M. Jepson R. (2014). A methodological systematic review of what’s wrong with meta-ethnography reporting. BMC Med. Res. Methodol. 14, 1471–2288. doi: 10.1186/1471-2288-14-119 PMC4277825 25407140

[ref38] Gangamma R. (2017). A phenomenological study of family experiences of resettled Iraqi refugees. J. Marital. Fam. Ther. 44, 323–335. doi: 10.1111/jmft.12251, PMID: 28677838

[ref39] Garcia M. F. Birman D. (2022). Understanding the migration experience of unaccompanied youth: a review of the literature. Am. J. Orthopsychiatry 92, 79–102. doi: 10.1037/ort0000588, PMID: 34881960

[ref40] Giddens A. (1984). The constitution of society: Outline of the theory of structuration. Cambridge: Polity Press.

[ref41] Green J. Thorogood N. (2018). Qualitative methods for health research. 4th Edn: Sage Publications.

[ref42] Haarlammert M. Birman D. Oberoi A. Moore W. J. (2017). Inside-out: representational ethics and diverse communities. Am. J. Community Psychol. 60, 414–423. doi: 10.1002/ajcp.12188 29027672

[ref43] Harunogullari M. Polat Y. (2017). Urban space experience and perceptions of Syrian refugee children [Conference]. 3rd International Scientific Conference GEOBALCANICA, Macedonia, Greece. Available online at: https://www.researchgate.net/deref/http%3A%2F%2Fdx.doi.org%2F10.18509%2FG.BP.2017.03 (accessed February 5, 2022).

[ref44] Hastings C. (2012). The experience of male adolescent refugees during their transfer and adaptation to a UK secondary school. Educ. Psychol. Pract. 28, 335–351. doi: 10.1080/02667363.2012.684342

[ref45] Hirad S. Miller M. M. Negash S. Lambert J. E. (2022). Refugee posttraumatic growth: a grounded theory study. Transcult. Psychiatry 60, 1–13. doi: 10.1177/13634615211062966 34994660

[ref46] Hodes M. Vasquez M. M. Anagnostopoulos D. Triantafyllou K. Abdelhady D. Weiss K. . (2018). Refugees in Europe: national overviews from key countries with a special focus on child and adolescent mental health. Eur. Child Adolesc. Psychiatry 27, 389–399. doi: 10.1007/s00787-017-1094-8, PMID: 29270786

[ref47] Horswood D. Baker J. Fazel M. Rees S. Heslop L. Silove D. (2019). School factors related to the emotional wellbeing and resettlement outcomes of students from refugee backgrounds: protocol for a systematic review. Syst. Rev. 8, 1–6. doi: 10.1186/s13643-019-1016-6 31039825 PMC6492402

[ref48] Including Children Affected by Migration (2022). Policy guidance on supporting inclusion of Ukrainian refugees in education. Available online at: https://www.icamproject.eu/good-news-for-the-icam-program/ (accessed August 21, 2022).

[ref49] Ioffe Y. Abubakar I. Issa R. Spiegel P. Kumar B. N. (2022). Meeting the health challenges of displaced populations from Ukraine. Lancet 399, 1206–1208. doi: 10.1016/S0140-6736(22)00477-9, PMID: 35286844 PMC8916778

[ref50] Jardim C. da Silva M. (2021). Belonging among young people with migrant background in Portugal: local, national, and transnational identifications. Soc. Ident. 27, 229–244. doi: 10.1080/13504630.2020.1816459

[ref51] Jarlby F. Derluyn I. Vitus K. Smith-Jervelund S. (2021). Attempts to “forget”: unaccompanied refugee adolescents' everyday experiences of psychosocial challenges and coping upon settlement. Int. J. Migr. Health Soc. Care 17, 181–195. doi: 10.1108/IJMHSC-04-2020-0030

[ref52] Jensen T. K. Bjørgo Skårdalsmo E. M. Fjermestad K. W. (2014). Development of mental health problems — a follow-up study of unaccompanied refugee minors. Child Adolesc. Psychiatry Ment. Health 8, 1–10. doi: 10.1186/1753-2000-8-29 25780387 PMC4361195

[ref53] Kardeş S. Kozikoğlu İ. (2021). Interactions between refugee and local preschool children and prejudice or discriminatory behaviours: teachers’ observations. J. Pedagog. Res. 5, 114–125. doi: 10.33902/JPR.2021067282

[ref54] Kastner M. Antony J. Soobiah C. Straus S. E. Tricco A. C. (2016). Conceptual recommendations for selecting the most appropriate knowledge synthesis method to answer research questions related to complex evidence. J. Clin. Epidemiol. 73, 43–49. doi: 10.1016/j.jclinepi.2015.11.022, PMID: 26912124

[ref55] Kia-Keating M. Ellis H. B. (2007). Belonging and connection to school in resettlement: young refugees, school belonging, and psychosocial adjustment. Clin. Child Psychol. Psychiatry 12, 29–43. doi: 10.1177/1359104507071052 17375808

[ref56] Korjonen-Kuusipuro K. Kuusisto A. K. Tuominen J. (2019). Everyday negotiations of belonging - making Mexican masks together with unaccompanied minors in Finland. J. Youth Stud. 22, 551–567. doi: 10.1080/13676261.2018.1523539

[ref57] Korpela M. (2016). A (sub)culture of their own? Children of lifestyle migrants in Goa, India. Asian Pac. Migr. J. 25, 470–488. doi: 10.1177/0117196816671959

[ref58] Kumi-Yeboah A. Brobbey G. Smith P. (2020). Exploring factors that facilitate acculturation strategies and academic success of west African immigrant youth in urban schools. Educ. Urban Soc. 52, 21–50. doi: 10.1177/0013124519846279

[ref59] Lähdesmäki T. Saresma T. Hiltunen K. Jäntti K. Sääskilahti N. Vallius A. . (2016). Fluidity and flexibility of “belonging”: uses of the concept in contemporary research. Acta Sociol. 59, 233–247. doi: 10.1177/0001699316633099

[ref60] Lee Y. M. Shin O. J. Lim M. H. (2012). The psychological problems of north Korean adolescent refugees living in South Korea. Psychiatry Investig. 9, 217–222. doi: 10.4306/pi.2012.9.3.217, PMID: 22993519 PMC3440469

[ref61] Lewin S. Booth A. Glenton C. Munthe-Kaas H. Rashidian A. Wainwright M. . (2018). Applying GRADE-CERQual to qualitative evidence synthesis findings: introduction to the series. Implement. Sci. 13, 1–70. doi: 10.1186/s13012-017-0688-3 29384079 PMC5791040

[ref62] Lewin S. Glenton C. Munthe-Kaas H. Carlson B. Colvin C. J. Gülmezoglu M. . (2015). Using qualitative evidence in decision making for health and social interventions: an approach to assess confidence in findings from qualitative evidence synthesis (GRADE-CERQual). PLoS Med. 12, 1–18. doi: 10.1371/journal.pmed.1001895 PMC4624425 26506244

[ref9001] Maadad N. Matthews J. (2017). Schooling Syrian refugees in Lebanon: Buildinghopeful futures. Educational Review 72, 459–474. doi: 10.1080/00131911.2018.1508126

[ref63] Majid U. Vanstone M. (2018). Appraising quantitative research for evidence synthesis: a compendium of quality appraisal tools. Qual. Health Res. 28, 2115–2131. doi: 10.1177/1049732318785358 30047306

[ref64] Majumder P. (2019). Exploring stigma and its effect on access to mental health services in unaccompanied refugee children. BJPsych Bull. 43, 275–281. doi: 10.1192/bjb.2019.35 31122304 PMC12402927

[ref65] Malsbary C. B.. (2013). The pedagogy of belonging: The social, cultural, and academic lives of recently-arrived immigrant youth in a multiethnic, multilingual high school. University of California: Dissertation Abstracts International, 73, 10-A(E).

[ref66] Malterud K. (2001). Qualitative research: standards, challenges, and guidelines. Lancet 358, 483–488. doi: 10.1016/S0140-6736(01)05627-6, PMID: 11513933

[ref67] Marcu S. (2012). Emotions on the move: belonging, sense of place and feelings identities among young Romanian immigrants in Spain. J. Youth Stud. 15, 207–223. doi: 10.1080/13676261.2011.630996

[ref68] Mayoma J. Williams Q. (2021). Fitting in: stylizing and (re)negotiating Congolese youth identity and multilingualism in Cape Town. Lingua 263, 102854–102813. doi: 10.1016/j.lingua.2020.102854, PMID: 40858408

[ref69] McCormack L. Tapp B. (2019). Violation and hope: refugee survival in childhood and beyond. Int. J. Soc. Psychiatry 65, 169–179. doi: 10.1177/0020764019831314 30808226

[ref70] McDiarmid S. Durbeej N. Sarkadi A. Osman F. (2021). Schools' and teachers' roles and challenges in supporting the mental wellbeing of refugee youths: a qualitative study with Swedish teachers. Int. J. Qual. Stud. Health Well-being 17, 1–15. doi: 10.1080/17482631.2021.2007568 PMC8648014 34847828

[ref71] Meloni F. (2019). The ambivalence of belonging: the impact of illegality on the social belonging of undocumented youth. Anthropol. Q. 92, 451–479. doi: 10.1353/anq.2019.0022

[ref72] Miller E. Ziaian T. Esterman A. (2018). Australian school practices and the education experiences of students with a refugee background: a review of the literature. Int. J. Incl. Educ. 22, 339–359. doi: 10.1080/13603116.2017.1365955

[ref73] Mitchell L. Bateman A. (2018). Belonging and culturally nuanced communication in a refugee early childhood Centre in Aotearoa New Zealand. Contemp. Issues Early Child. 19, 379–391. doi: 10.1177/1463949118781349

[ref74] Moensted M. L. (2020). Nearness and distance: the double-sided nature of belonging for young refugees in Australia. J. Study Race Natl. Cult. 26, 270–285. doi: 10.1080/13504630.2020.1761316

[ref75] Moher D. Liberati A. Tetzlaff J. Altman D. Antes G. Atkins D. . (2009). Preferred reporting items for systematic review and meta-analyses: the PRISMA statement. PLoS Med. 6:97. doi: 10.1371/journal.pmed.1000097 PMC2707599 19621072

[ref76] Moher D. Shamseet L. Clarke M. Ghersi D. Liberati A. Petticrew M. . (2015). Preferred reporting items for systematic review and meta-analysis protocols (PRISMA-P) 2015 statement. Syst. Rev. 4, 1–9. 25554246 10.1186/2046-4053-4-1 PMC4320440

[ref77] National Institute of Health and Care Excellence (2012). Supplementary materials H quality appraisal checklist – Qualitative studies: Methods for the development of NICE public health guidance (third edition): Guidance. Available online at: https://www.nice.org.uk/process/pmg4/chapter/Supplementary%20Materials-h-quality-appraisal-checklist-qualitative-studies (accessed May 8, 2022).

[ref78] Ndofor-Tah C. Strang A. Phillimore J. Morrice L. Michael L. Wood P. . (2019). Home office indicators of integration framework 2019. Available online at: https://assets.publishing.service.gov.uk/government/uploads/system/uploads/attachmentdata/file/1074688/home-office-indicators-of-integration-framework-2019-horr109.pdf

[ref79] NHS England (2018). Guidance for commissioners: Interpreting and translation services in primary care. Available online at: https://www.england.nhs.uk/wp-content/uploads/2018/09/guidance-for-commissioners-interpreting-and-translation-services-in-primary-care.pdf (accessed August 2, 2022).

[ref80] Ni Raghallaigh M. Gilligan R. (2010). Active survival in the lives of unaccompanied minors: coping strategies, resilience, and the relevance of religion. Child Family Social Work 15, 226–237. doi: 10.1111/j.13652206.2009.00663.x

[ref81] Noblit G. W. Hare R. D. (1988). Meta-ethnography: Synthesising qualitative studies. Newbury Park: SAGE Publications, Inc.

[ref82] Nunn C. Spaaij R. Luguetti C. (2022). Beyond integration: football as a mobile, transnational sphere of belonging for refugee-background young people. Leis. Stud. 41, 42–55. doi: 10.1080/02614367.2021.1962393

[ref83] Nye E. Melendez-Torres G. J. Bonell C. (2016). Origins, methods and advances in qualitative meta-synthesis. Rev. Educ. 4, 57–79. doi: 10.1002/rev3.3065

[ref84] Office for Health Improvement and Disparities (2017). Guidance. Mental health: Migrant health guide. Advice and guidance on the health needs of migrant patients for healthcare practitioners. Available online at: https://www.gov.uk/guidance/mental-health-migrant-health-guide (accessed August 29, 2022).

[ref85] Orupabo J. Drange I. Abrahamsen B. (2019). Multiple frames of success: how second-generation immigrants experience educational support and belonging in higher education. High. Educ. 79, 921–937. doi: 10.1007/s10734-019-00447-8

[ref86] Osman F. Mohamed A. Warner G. Sarkadi A. (2020). Longing for a sense of belonging–Somali immigrant adolescents’ experiences of their acculturation efforts in Sweden. Int. J. Qual. Stud. Health Well-being 15, 1–13. doi: 10.1080/17482631.2020.1784532 PMC7734117 33297899

[ref87] Pastoor L. (2015). The mediational role of schools in supporting psychosocial transitions among unaccompanied young refugees upon resettlement in Norway. Int. J. Educ. Dev. 41, 245–254. doi: 10.1016/j.ijedudev.2014.10.009

[ref88] Pieloch K. McCullough M. B. Marks A. K. (2016). Resilience of children with refugee statuses: a research review. Can. Psychol. 57, 330–339. doi: 10.1037/cap0000073

[ref89] Pollard T. Howard N. (2021). Mental healthcare for asylum-seekers and refugees residing in the United Kingdom: a scoping review of policies, barriers, and enablers. Int. J. Ment. Health Syst. 15, 1–15. doi: 10.1186/s13033-021-00473-z 34127043 PMC8201739

[ref90] Radjack R. Touhami F. Di C. Mouchenik Y. Minassian S. Moro M. R. (2021). Transition to the majority of unaccompanied minors: what adaptations are necessary for psychological management and the transcultural clinic? Ann. Medico Psychol. 179, 173–180. doi: 10.1016/j.amp.2020.03.004

[ref91] Reinelt T. Vasileva M. Petermann F. (2016). Refugee children’s mental health problems: beyond posttraumatic stress disorder. Kindheit Entw. 25, 231–237. doi: 10.1026/0942-5403/a000207

[ref92] Renzaho A. M. N. Dhingra N. Georgeou N. (2017). Youth as contested sites of culture: the intergenerational acculturation gap amongst new migrant communities-parental and young adult perspectives. PLoS One 12, 1–19. doi: 10.1371/journal.pone.0170700 PMC5295684 28170406

[ref93] Richardson W. S. Wilson M. C. Nishikawa J. Hayward R. S. (1995). The well-built clinical question: a key to evidence-based decisions. ACP J. Club 123, A12–A13. doi: 10.7326/ACPJC-1995-123-3-A12 7582737

[ref94] Rodriguez I. M. Dobler V. (2021). Survivors of hell: resilience amongst unaccompanied minor refugees and implications for treatment – a narrative review. J. Child Adolesc. Trauma 14, 559–569. doi: 10.1007/s40653-021-00385-7, PMID: 34820043 PMC8586295

[ref95] Ruth A. Estrada E. Martinez-Fuentes S. Vazquez-Ramos A. (2019). Soy de aquí,soy deallá: DACAmented homecomings and implications for identity and belonging. Latino Stud. 17, 304–322. doi: 10.1057/s41276-019-00197-9

[ref96] Rutter J. (2003). Working with refugee children. York: Joseph Rowntree Foundation.

[ref97] Salami B. Fernandez-Sanchez H. Fouche C. Evans C. Sibeko L. Tulli M. . (2021). A scoping review of the health of African immigrant and refugee children. Int. J. Environ. Res. Public Health 18:3514, 1–21. doi: 10.3390/ijerph18073514 33800663 PMC8038070

[ref98] Sattar R. Lawton R. Panagioti M. Johnson J. (2021). Meta-ethnography in healthcare research: a guide to using a meta-ethnographic approach for literature synthesis. BMC Health Serv. Res. 21, 1–13. doi: 10.1186/s12913-020-06049-w 33419430 PMC7796630

[ref99] Schachner M. K. Noack P. Van de Vijver F. J. Eckstein K. (2016). Cultural diversity climate and psychological adjustment at school— equality and inclusion versus cultural pluralism. Child Dev. 87, 1175–1191. doi: 10.1111/cdev.12536 27091829

[ref100] Scutaru B. (2021). Childhood memories of belonging among young Romanian migrants in Italy: a qualitative life-course approach. Childhood 28, 409–426. doi: 10.1177/09075682211033018

[ref101] Shmulyar Gréen O. Melander C. Höjer I. (2021). Identity formation and developing meaningful social relationships: the role of the polish Catholic community for polish young people migrating to Sweden. Front. Psychol. 6:638. doi: 10.3389/fsoc.2021.660638, PMID: 34026902 PMC8138302

[ref102] Sime D. (2020). New scots? Eastern European young people’s feelings of belonging and national identity in Scotland post-Brexit. Scott. Aff. 29, 336–353. doi: 10.3366/scot.2020.0327

[ref103] Sime D. Fox R. (2015). Migrant children, social capital and access to services post-migration: transitions, negotiations and complex agencies. Child. Soc. 29, 524–534. doi: 10.1111/chso.12092

[ref104] Sobitan T. (2022). Understanding the experiences of school belonging amongst secondary school students with refugee backgrounds (UK). Educ. Psychol. Pract. 38, 259–278. doi: 10.1080/02667363.2022.2084042

[ref105] Sone S. Thang L. L. (2020). Staying till the end? Japanese later-life migrants and belonging in Western Australia. Jpn. Stud. 40, 41–62. doi: 10.1080/10371397.2020.1712998

[ref106] Tartakovsky E. (2009). The psychological well-being of unaccompanied minors: a longitudinal study of adolescents immigrating from Russia and Ukraine to Israel without parents. J. Res. Adolesc. 19, 177–204. doi: 10.1111/j.1532-7795.2009.00589.x

[ref107] Toye F. Seers K. Allcock N. Briggs M. Carr E. Barker K. (2014). Meta-ethnography 25 years on: challenges and insights for synthesising a large number of qualitative studies. BMC Med. Res. Methodol. 14, 1471–2288. doi: 10.1186/1471-2288-14-80 PMC4127190 24951054

[ref108] United Nations High Commissioner for Refugees. (2022a). Figures at a glance. UNHCR: The UN Refugee Agency. Available online at: https://www.unhcr.org/figures-at-a-glance.html (accessed February 2, 2022).

[ref109] United Nations High Commissioner for Refugees. (2022b) Refugee data finder. UNHCR: The UN Refugee Agency. Available online at: https://www.unhcr.org/refugee-statistics/download/?url=aU4R9J (Accessed February 2, 2022).

[ref110] Uptin J. (2021). ‘If I peel off my black skin maybe then I integrate’. Examining how African-Australian youth find living in a ‘post multicultural’ Australia. J. Study Race Natl. Cult. 27, 75–91. doi: 10.1080/13504630.2020.1814726

[ref111] Van Os E. C. C. Zijlstra A. E. Knorth E. J. Post W. J. Kalverboer M. E. (2020). Finding keys: a systematic review of barriers and facilitators for refugee children’s disclosure of their life stories. Trauma Violence Abuse 21, 242–260. doi: 10.1177/1524838018757748 29463187 PMC7016356

[ref112] Weine S. M. Ware N. Hakizimana L. Tugenberg T. Currie M. Dahnweih G. . (2014). Fostering resilience: protective agents, resources, and mechanisms for adolescent refugees’ psychosocial wellbeing. Adolesc. Psychiatry 4, 164–176. doi: 10.2174/221067660403140912162410, PMID: 25544939 PMC4274391

[ref113] Welply O. (2015). Re-imagining otherness: an exploration of the global imaginaries of children from immigrant backgrounds in primary schools in France and England. Eur. Educ. Res. J. 14, 430–453. doi: 10.1177/1474904115603733

[ref114] Wilding R. (2009). Refugee youth, social inclusion, and ICTs: can good intentions go bad? J. Inf. Commun. Ethics Soc. 7, 159–174. doi: 10.1108/14779960910955873

[ref115] Wille A. M. Maher M. K. Cornell S. R. Kim A. C. Reimers B. Hess R. S. (2019). It starts with us: including refugees in rural schools and communities. Rural. Educ. 40, 33–42. doi: 10.35608/ruraled.v40i2.850

[ref116] Wofford M. C. Tibi S. (2018). A human right to literacy education: implications for serving Syrian refugee children. Int. J. Speech Lang. Pathol. 20, 182–190. doi: 10.1080/17549507.2017.1397746, PMID: 29171285

[ref117] Woodgate R. L. Busolo D. S. (2018). Above chaos, quest, and restitution: narrative experiences of African immigrant youth’s settlement in Canada. BMC Public Health 18, 1–12. doi: 10.1186/s12889-018-5239-6, PMID: 29514615 PMC5842655

[ref118] Zayas L. H. Gulbas L. E. (2017). Processes of belonging for citizen-children of undocumented Mexican immigrants. J. Child Fam. Stud. 26, 2463–2474. doi: 10.1007/s10826-017-0755-z, PMID: 30233124 PMC6141042

[ref119] Zeynep I. (2012). In pursuit of a new perspective in the education of children of the refugees: advocacy for the “family”. Educ. Sci. Theory Pract. 12:3025.

