# Supplementary material for: First-hand experiences of belonging among child refugees and asylum seekers, post-migration: a meta-synthesis
Source: Front Psychol. 2025 Oct 22;16:1603733. doi: 10.3389/fpsyg.2025.1603733 (PMC12586878; doi:10.3389/fpsyg.2025.1603733)
Supplement: Supplementary file 1 [file Table_1.DOCX]

**Supplementary Materials A**

Defining Refugees

The term ‘refugee’ is highly disputed, with some suggesting that it is used by host countries as a means of separating and containing those defined by the term, while others view it as more of an administrative label used in place of a lost identity (Crawford, 2017). In actuality, being a refugee or asylum seeker is not a lifelong identity, but a transitional process; however, the term can be politically and socially constructed by others (e.g., the government, policies, or through media stereotypes), and can often be imposed on displaced people who may have stopped identifying themselves as refugees long before others do.

Generally speaking, ‘refugee’ is intended to describe those who have involuntarily migrated and are resettled in a country where the government has agreed to accept them. The term asylum seeker describes those who are still undergoing the formal process of seeking refuge and protection in a new country, typically due to fear of persecution in their home country (Hastings, 2012; Kumi-Yeboah et al., 2020).

Young refugees have been described as “victims of their own past” (Korjonen- Kuusipuro et al., 2019, p.551); a label which can go on to define their care needs in numerous institutional settings, from school to the care system or social services. UASC in particular often live in an open-ended ‘limbo’ state of being and waiting for several years. It is well documented that these young people have limited opportunities to live like their non-refugee peers, who are unlikely to relate to the overwhelming and uncertain experience of having to flee their home and seek asylum in a new country. These experiences are commonly linked to loneliness, post-traumatic stress, and other psychological conditions (Reinelt et al., 2016; Van os et al., 2020).

Table B1

*PICOS Model*

**P** Population, patient, or problem (e.g., age, gender, disorder/disease)

**Supplementary Materials B**

Child refugees or asylum seekers, including UASC, resettled in any host country that is not their country of origin. Child refugees and asylum seekers are defined as children, aged 18 years old and under, who have migrated to other countries due to fear of persecution in their country of origin due to factors such as race, religion, nationality, or political opinion (UNHCR, 2022b).

**I** Intervention, exposure, variable(s) of interest, or risk/prognostic factor(s)

This review will focus on research that attempts to describe the belonging experiences of children who have been exposed to migration/resettlement, as opposed to a specific intervention. These experiences might be described in the context of relationships with family/peers/wider systems within their host country, or the new cultural ‘norms’ and expectations they are adjusting to.

**C** Comparators/comparison or control

Predominantly interested in belonging experiences, rather than comparing outcomes between groups. Therefore, this review will not involve a control group. Studies that have used control groups in their efforts to enhance understanding of belonging experiences will be considered for review.

**O** Outcome(s) The primary outcome is a sense of belonging. This will be assessed via first-hand accounts gathered through self-report measures (e.g., semi-structured interviews, focus groups). As belonging is a fluid concept, a range of terms that capture its essence are felt to be necessary when conducting the search. Search terms will be simple and broad, to capture all potentially relevant studies.

**S** Study design/type Due to the exploratory nature of this review and interest in lived experiences, a qualitative approach is considered most appropriate. Analysis will be in the form of a meta-synthesis, guided by meta- ethnography, guided by Noblit and Hare’s (1988) steps.

*Note.* Introduced by Richardson et al. (1995), updated by Moher et al. (2009)

**Supplementary Materials C**


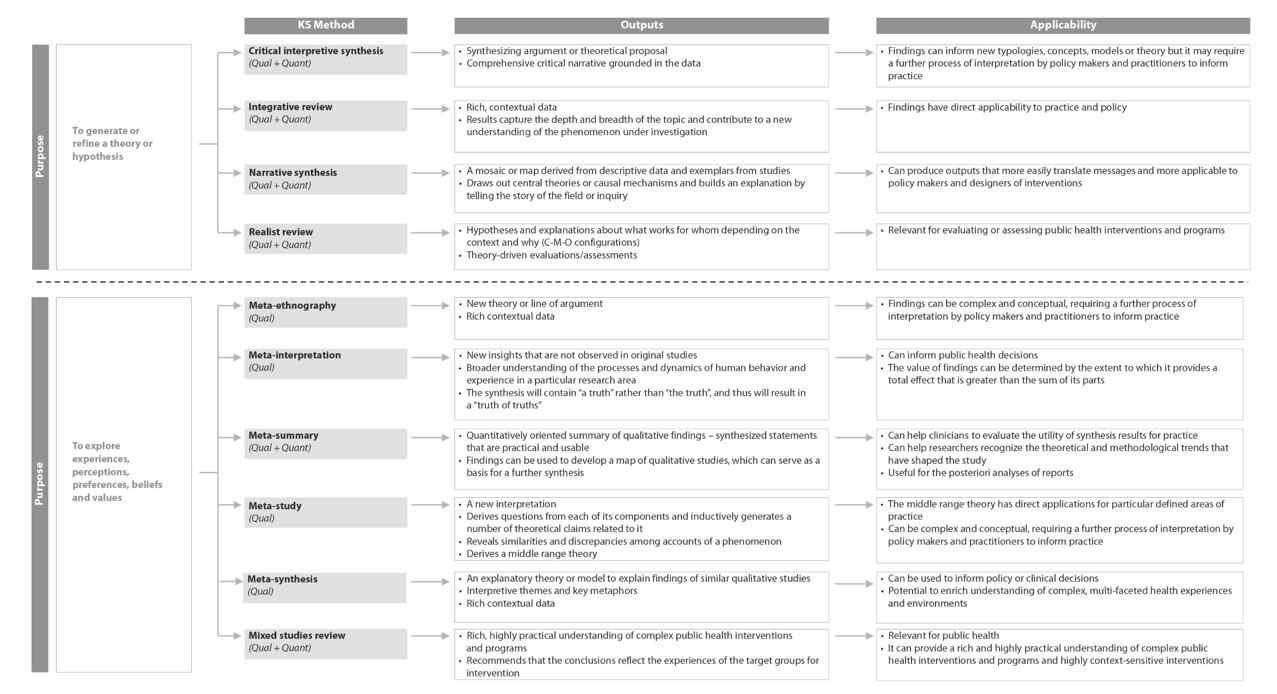


Figure C1

*Conceptual algorithm to optimise selection of a knowledge synthesis method for answering a research question (Kastner et al., 2016)*

**Supplementary Materials D**

Screen Captures of Study Screening Process

Figure D1

*Screen Capture of Initial Title Level Screen*


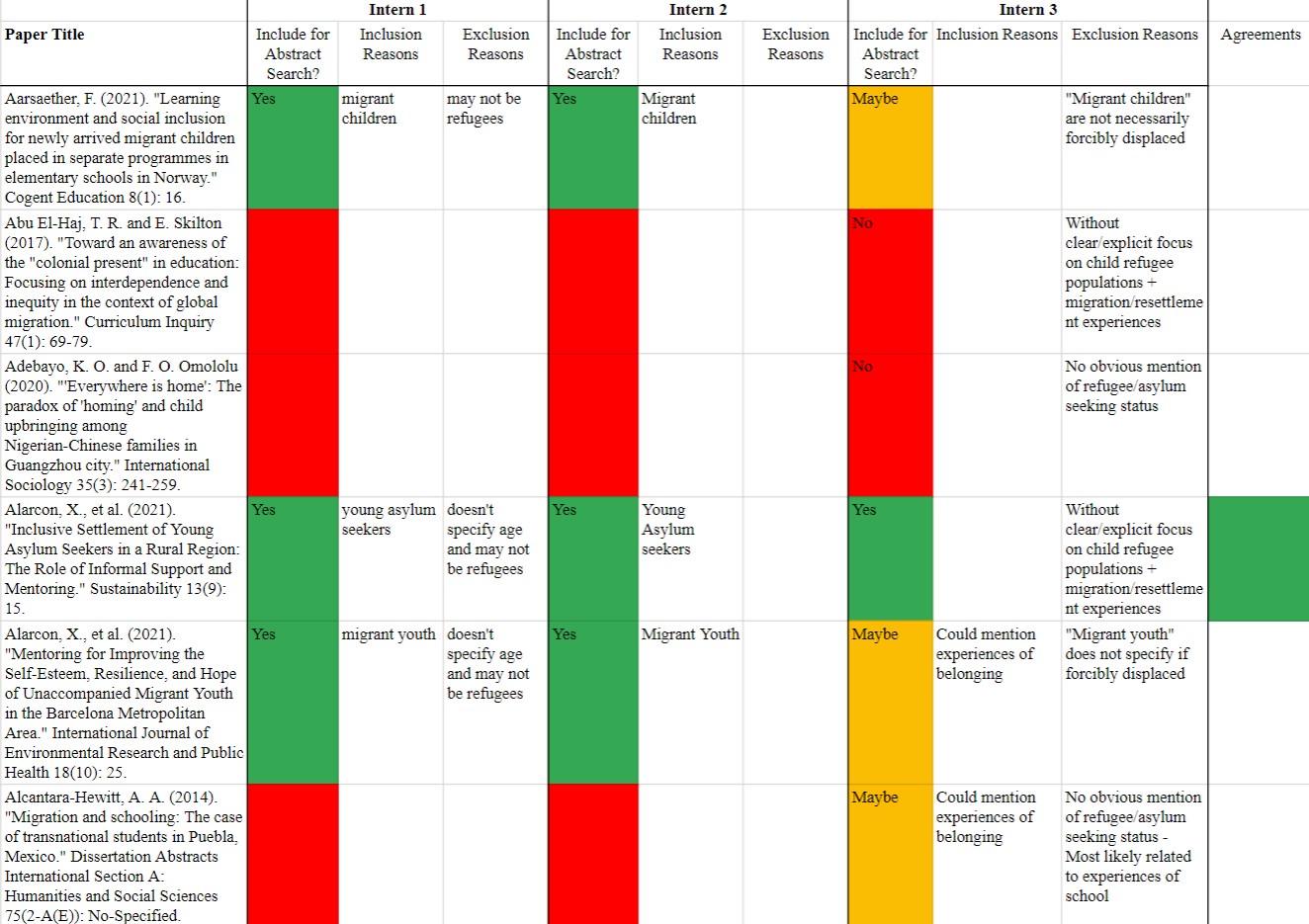


*Note.* ‘Traffic light’ system to indicate individual decisions to include a study (green), exclude (red), or seek further information (amber) via an abstract or full text screen, or further consultation with the group.

Figure D2

*Screen Capture of Full Text Screen*


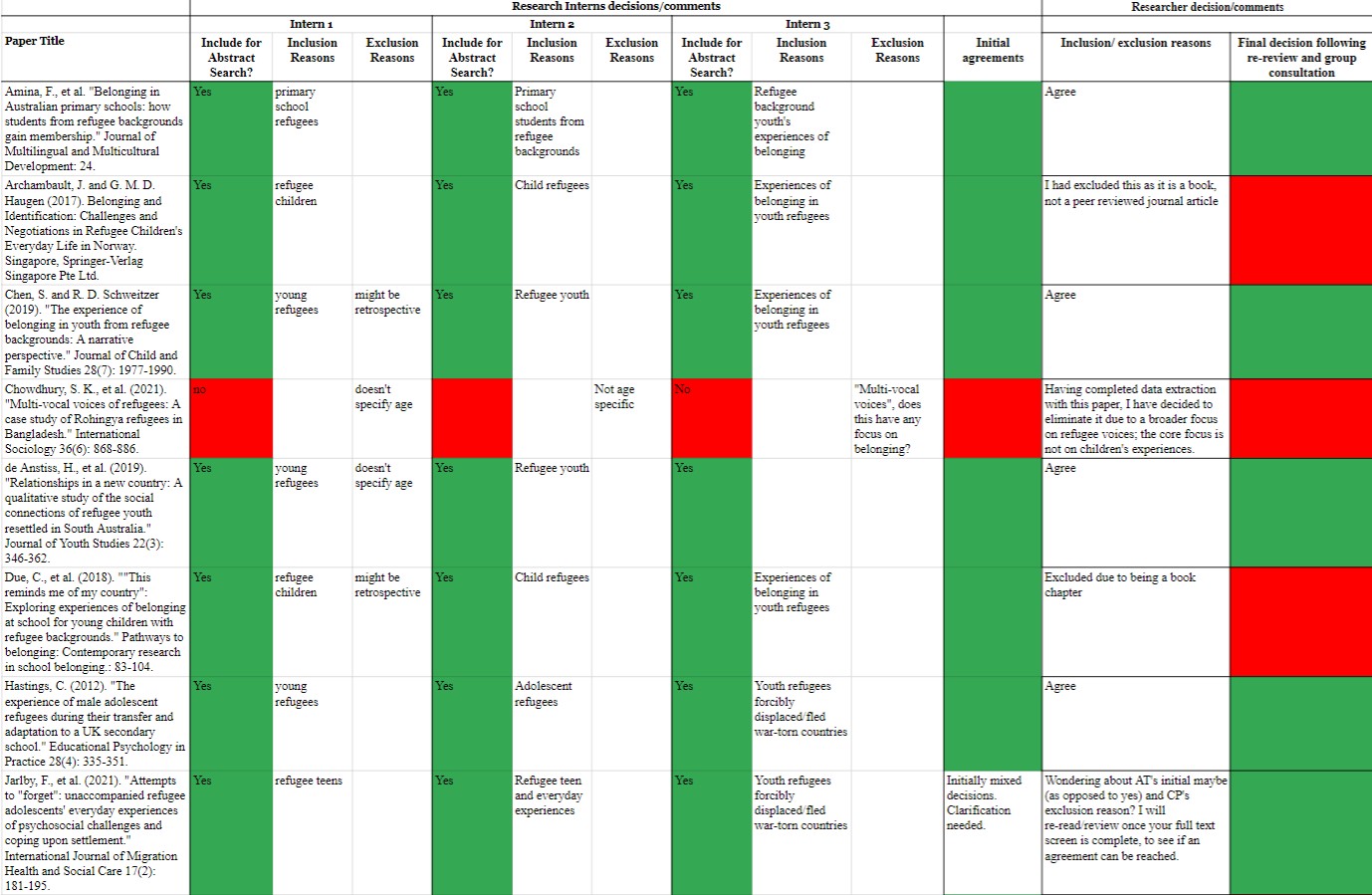


**Supplementary Materials E**

Table E1

*18-item Integrated Quality Appraisal Checklist*

Checklist item Sub questions

1. Are funding and/or sources of conflict clearly acknowledged?
2. Is there an abstract? *Does the abstract adequately describe the study's aims, design,*

*and main implications and/or significance? Does the abstract state the question/objective?*

*Does the abstract adequately describe the study design? Does the abstract list the analytic strategy?*

*Does the abstract list the main implications and/or significance?*

1. Theoretical approach:

Is a qualitative approach appropriate?

1. Theoretical approach:

Is the study clear in what it seeks to do?

*Is a rationale given for using a qualitative approach?*

*Is it clear how prior understandings of the phenomena under study were managed and/or influenced the research?*

*Does the study have a clear rationale and aim? Is the approach to inquiry clear?*

1. Ethics *How clear and coherent is the reporting of ethics?*
2. Concepts:

Is belonging defined?

1. Concepts:

Is refugee/asylum seeker/unaccompanied child, etc. defined?

1. Study design:

How defensible/rigorous is the research design/methodology?

*Is the choice of method clearly described?*

*Is the design appropriate to the research question?*

*Are there clear accounts of the rationale/justification for data collection and data analysis techniques used?*

*Is the selection of cases/sampling strategy*

Checklist item Sub questions

*theoretically justified?*

*Are intended participants clearly defined? Is the recruitment process clear?*

1. Data collection:

How well was the data collection carried out?

1. Trustworthiness:

Is the role of the researcher clearly described?

1. Trustworthiness:

Is the context clearly described?

1. Trustworthiness:

Were the methods reliable?

1. Analysis:

Is the data sufficiently rigorous?

1. Analysis:

Is the data rich?

*Is the data collection strategy clear?*

*Were the appropriate data collected to address the research question?*

*Were the mean and range of time duration for interviews and other data collection tools clear/appropriate?*

*Is the procedure reliable/dependable?*

*Were coders and analysts and their training clearly described? Is the role of the researcher clearly described?*

*Does the paper describe how the research was explained and presented to the participants?*

*Has the relationship between the researcher and the participants been adequately considered?*

*Was researcher bias considered?*

*Are sampled participants clearly defined?*

*Are the demographics and/or cultural information, perspectives of participants, or characteristics of data clear and appropriate?*

*Was data collected by more than 1 method? Do the methods investigate what they claim to?*

*Is the process of analysis clear?*

*Is it clear how the themes and concepts were derived from the data?*

*Is the procedure reliable/dependable?*

*Has the researcher discussed contradictory data (or suggested why there is none)?*

*How well has the detail and depth been demonstrated?*

*Are responses compared and contrasted across groups/sites?*

1. Analysis: *Were softwares that were used indicated?*

Checklist item Sub questions

Is the analysis reliable? *Did more than 1 researcher theme and code transcripts/ data?*

*Did the researchers make clear how differences, if at all, would have been resolved?*

1. Results:

Are the findings convincing?

1. Results:

Are the findings relevant to the aims of the study?

1. Conclusions:

Are the conclusions adequate?

*Is the reporting clear and coherent? Are the findings convincing?*

*Were findings clearly presented?*

*Are the research findings compatible with the study design? Are extracts from the original data included?*

*Were the findings grounded in the evidence?*

*Did the data adequately capture forms of diversity most relevant to the question, research goals, and inquiry approach?*

*Were similarities and differences from prior theories and research findings identified?*

*Did researchers make clear how findings were or can be best utilised?*

*Were any reflections made on alternative findings?*

*Have alternative explanations been explored and discounted? Does this enhance understanding of the research topic?*

*Note.* Adapted from APA (2018) JARS-Qual. and NICE (2012) guidance.

145

Figure E1

*Screen Capture of Quality Appraisal Process*


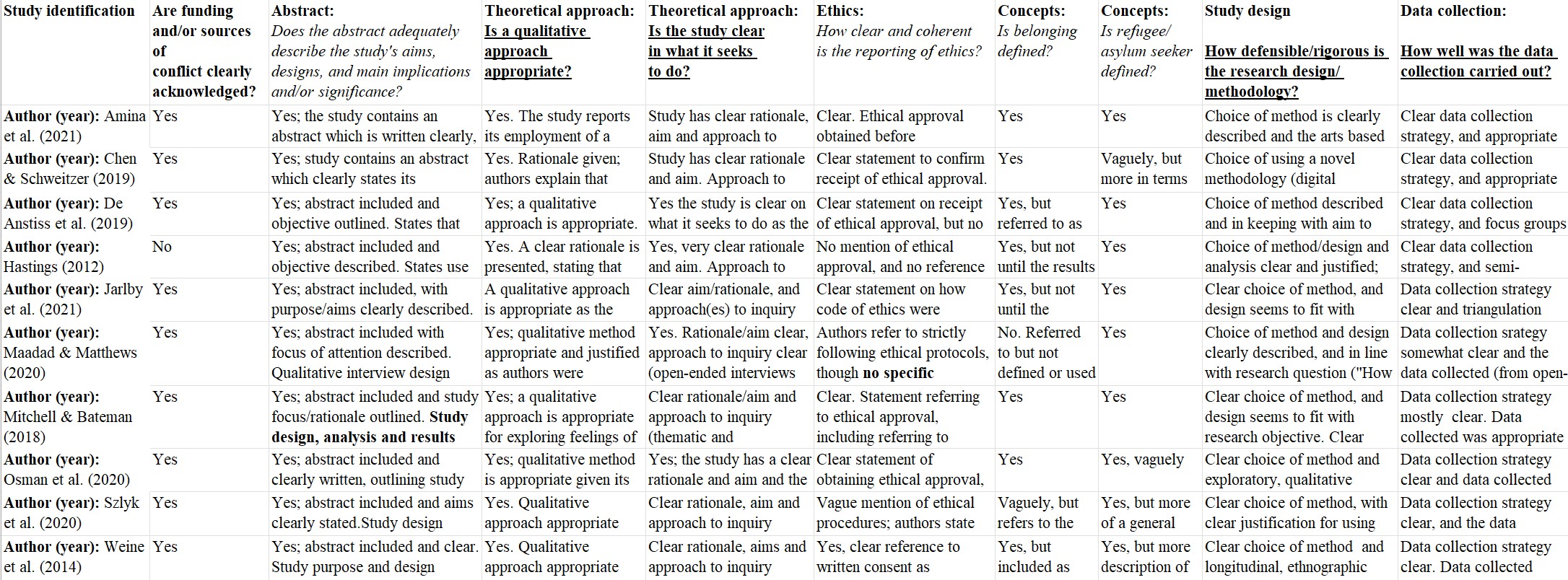


**Supplementary Materials F**

Reducing Themes into Key Conceptual Categories

1. **Concept of support and inclusion**
2. **Concept of exclusion**
3. **Seeking protection**
4. **Helping others**
5. **Family connectedness**
6. **Severed relationships & disconnection**
7. **Shared activities**
8. **Recommendations for teachers**
9. **Concept of language and culture: facilitators *and* barriers to belonging?**
10. **Financial burden**
11. **Maintaining connections to country of origin**
12. **Recreating feelings of family and home**
13. **Belonging to a shared earth**
14. **Concept of gratitude**
15. **Migratory loss and loneliness**
16. **Coping strategies**
17. **Stigma, discrimination & societal isolation**
18. **Gender inequalities**
19. **A sense of identity**
20. **Distrust of services**
21. **Physical and emotional impact of resettlement**
22. **Thinking about the future**
23. **Being seen (added by reviewer 1)**
24. **Being infantilised, underestimated and dehumanised (added by reviewer 1)**
25. **Words of encouragement (added by reviewer 2)**

**Supplementary Materials G**

Translating Studies Into One Another

Findings from study one (Amina et al., 2021) show that both peers and teachers can support the process of belonging for newly resettled primary school children. This was facilitated through offering support with language development, inclusion in games by peers or in class discussions by teachers. For some, inclusion fostered feelings of safety and protection, too. On the other hand, these same facilitators to belonging could also serve as barriers, with some participants describing experiences of being excluded by other children, feeling unable to enjoy activities due to the language barrier, or feeling “invisible” to teachers, when compared to their non-refugee peers. The children’s specific cultural practices and financial circumstances were also factors in their resettlement, with some referring to not being able to afford food in the canteen or find the appropriate food for their culture.

Similarly, study two (Osman et al., 2020) found that support from teachers was essential for their adjustment to a new school. However, with its focus being on secondary school and participants who migrated during their adolescent years, many referred to it being harder to resettle. This was attributed to finding it harder to learn the language than if they had migrated at a younger age, experiences of being discriminated at school, and a feeling of falling in- between cultures. A recurring theme was the lack of adult support available to participants, both at home and at school, differing somewhat from the first study. This study also went beyond the remit of study one by exploring coping strategies that helped children adapt to a new culture, including acceptance, hope, and gratitude for the opportunities they now have, which contrast with how life might have been if they had remained in their war-torn home countries. They also took responsibility for creating integration experiences, suggesting a sense of agency that the primary school participants in study one did not refer to.

Correspondingly, study three (Chen & Schweitzer, 2019) took place within a school setting, and found the support of others, namely teachers, and help with learning English, to be important for a sense of belonging. Comparable to study one, feelings of safety and protection through inclusion were also mentioned. However, this study differed from the previous two in terms of the participant accounts that alluded to belonging to a larger entity (the earth, nature, the present moment, past memories, objects, spirituality, etc.). A similarity between this study and the first two studies was the polarised accounts of participants. For some, relational bonds (e.g., friends and family) underpinned the process of belonging to a new school and culture, but for others, who had been separated from friends and family, the feelings of loss and yearning for these people served as a barrier to belonging.

Study four (Jarlby et al., 2021) differed from the previous studies in terms of context, a residential setting. Like previous studies, it spoke to the importance of building connections and engaging in shared activities to build a sense of belonging. However, it also described instances of social withdrawal in response to language barriers and perceived stigma, thus positioning themselves as ‘outsiders’. Feelings of stigma, difference and loneliness were referred to more than in any other study, with the metaphor of a boat at sea being used to describe being alone. As with other studies, these factors served as potential barriers to belonging. This study also referred to experiences of societal exclusion (e.g., prejudices and racism).

Study five (de Anstiss et al., 2019) differed from studies 1-4 due to being based in the local community. However, like the previous studies, it highlighted the importance of family, peer and community support, only with more emphasis on the voices of those experiencing a *lack* of support. Experiences of intense conflict, abuse and violence reported and attributed to the stress of displacement, and parents struggling to belong while their children adjust to their new culture (creating a distance within families). This stirred up references to bicultural identities, similar to study two. Like study one, participants within this study spoke more about their financial circumstances and the effect of these on their ability to resettle. Unlike the previous studies, study five commented on a general distrust towards service providers due to fears of being misunderstood. Gender roles and inequalities were also referenced in this study.

Study six (Mitchell & Bateman, 2018), like studies 1-3, took place in an early childhood education centre, and predominantly focused on welcoming newly resettled children; thus supporting the concept of support and inclusion. This was facilitated through shared activities and attempts to maintain participants’ home country connections (e.g., by speaking to them in their home language, using greetings from their culture).

Study seven (Weine et al., 2014) similarly noted the importance of support (‘protective agents) from family members, refugee communities, church, school, and the local community. Barriers to belonging were not alluded to.

Study eight (Hastings, 2012) also found help from teachers, peers and family to be of the utmost importance, especially during the beginning of resettlement. As with studies one, three, and seven, themes of seeking safety and protection were noted, especially in relation to bullying experiences. The paper concluded that peers may also be a barrier, as well as facilitator, to belonging experiences. In addition, a finding shared with all previous studies was that learning the English seemed to support the development of belonging in their host country.

Table G1

*Collaborative Translation of Studies*


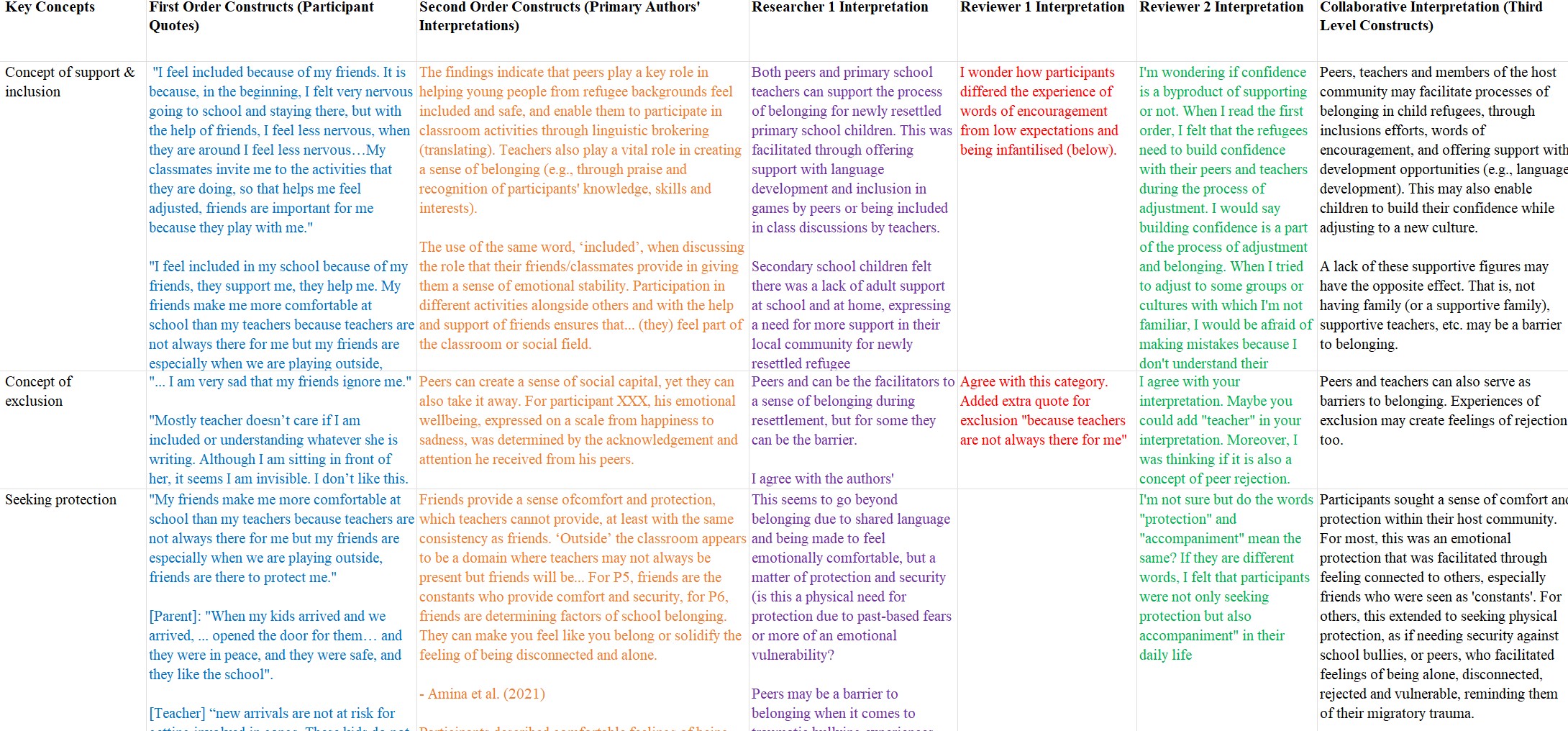


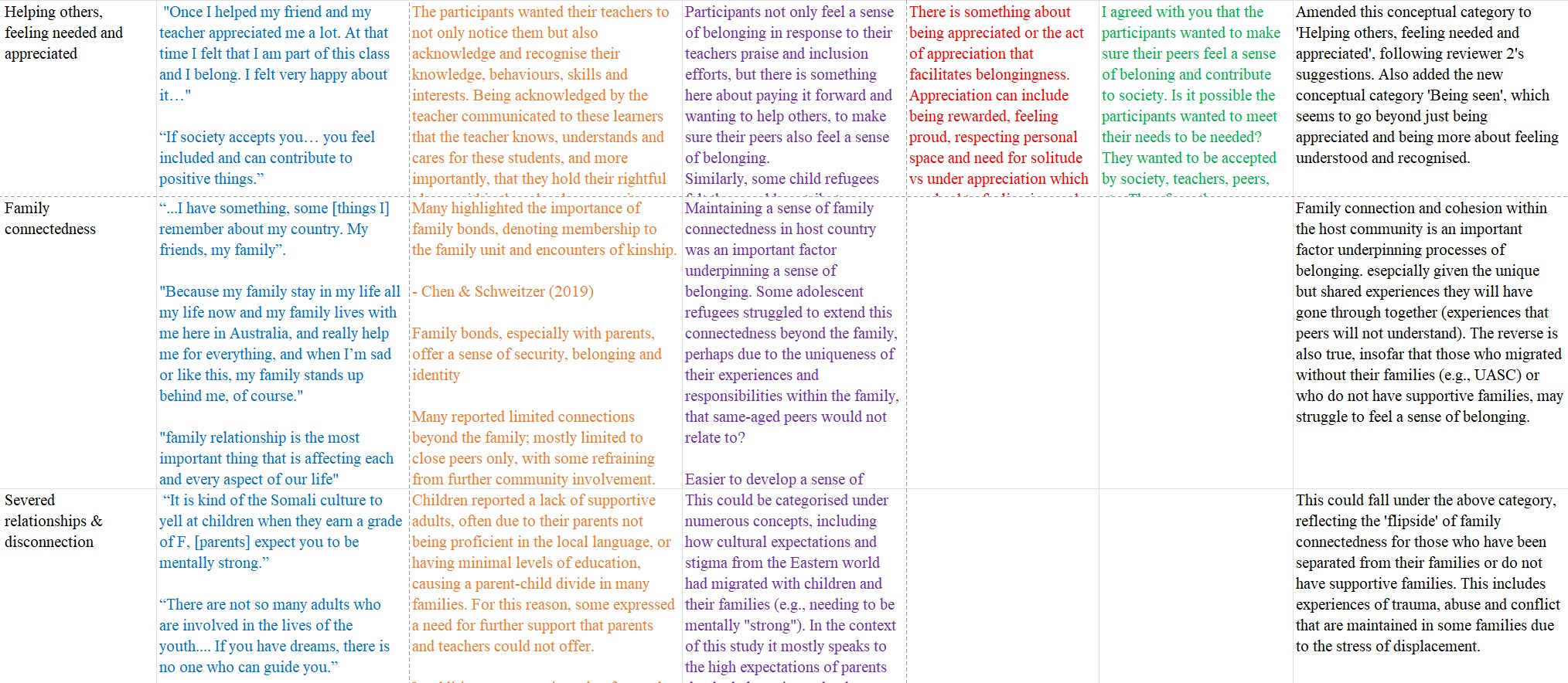


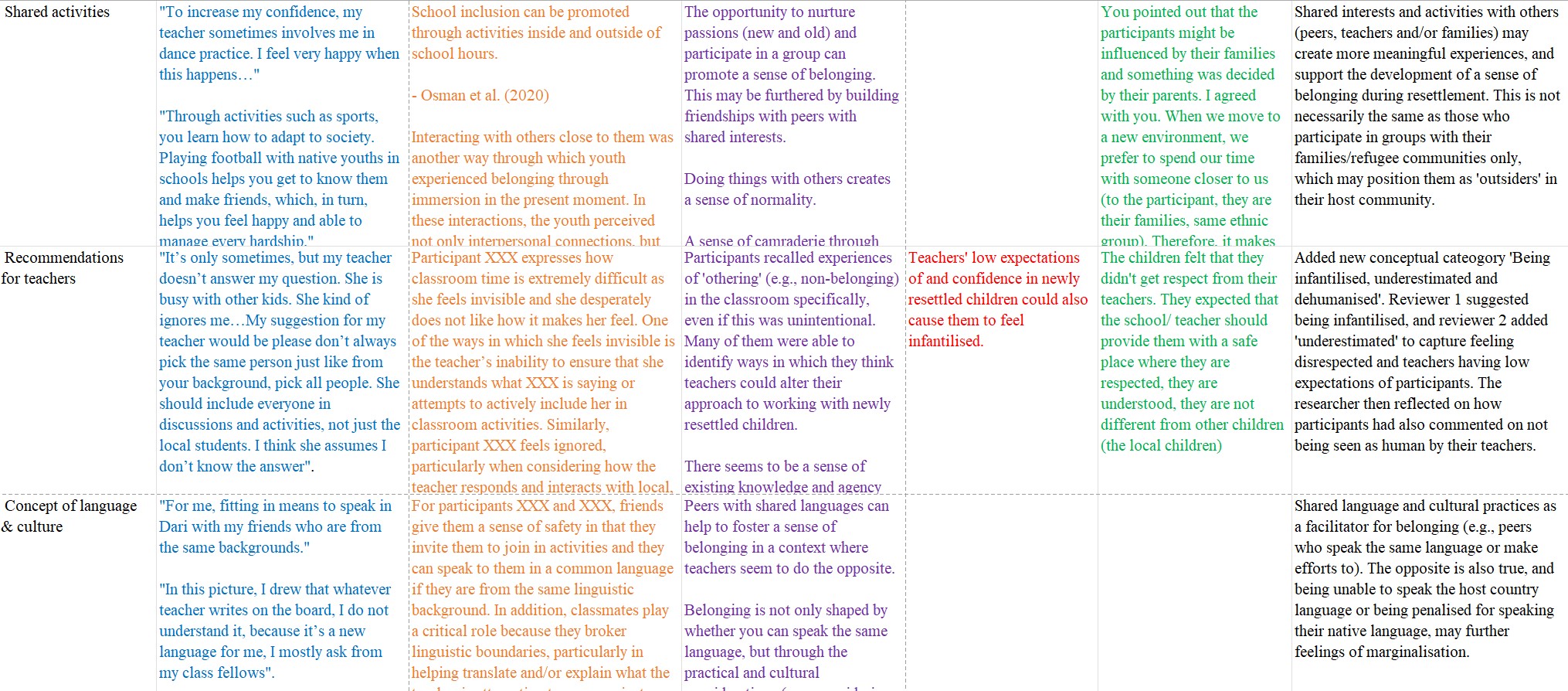


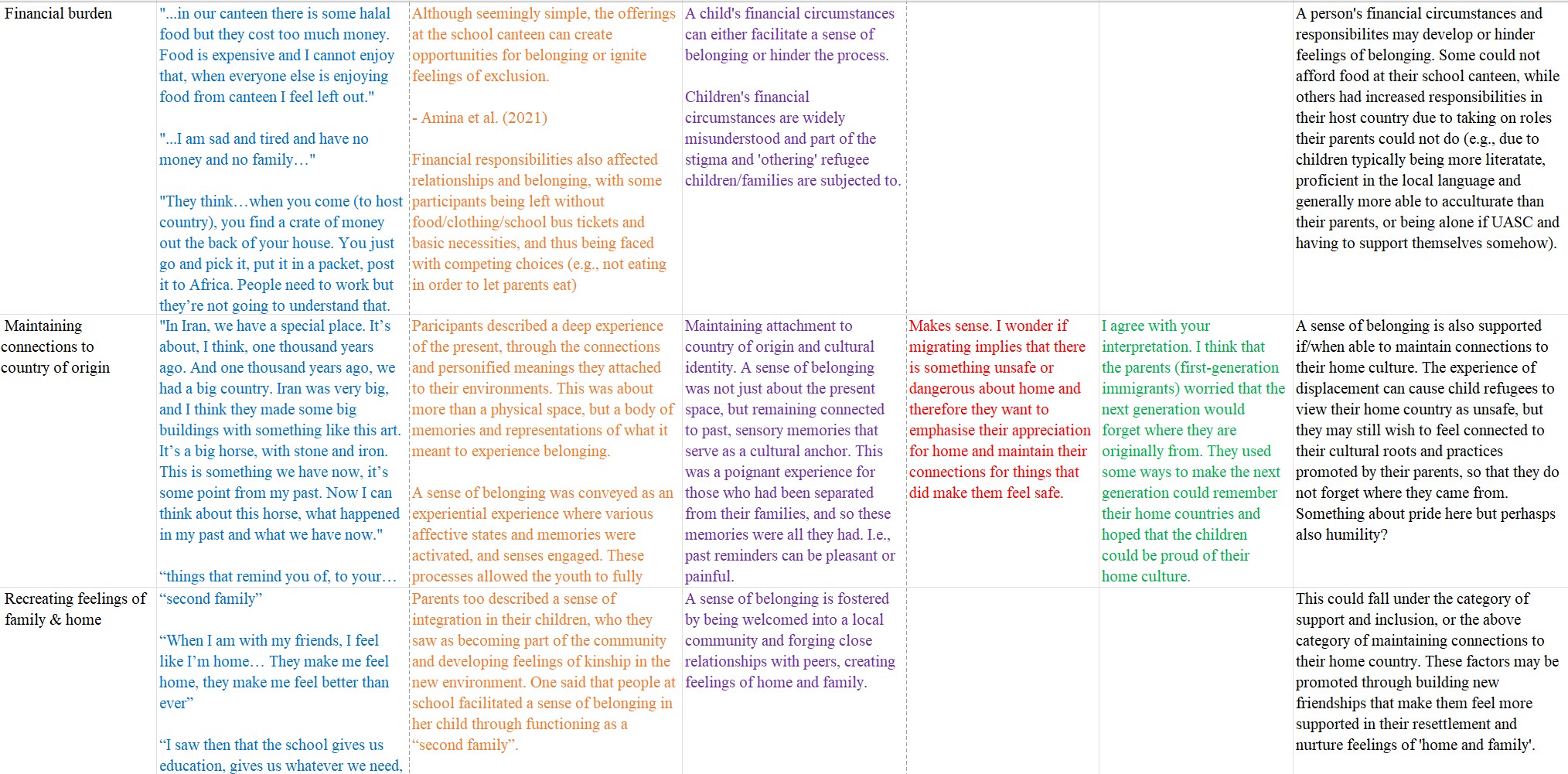


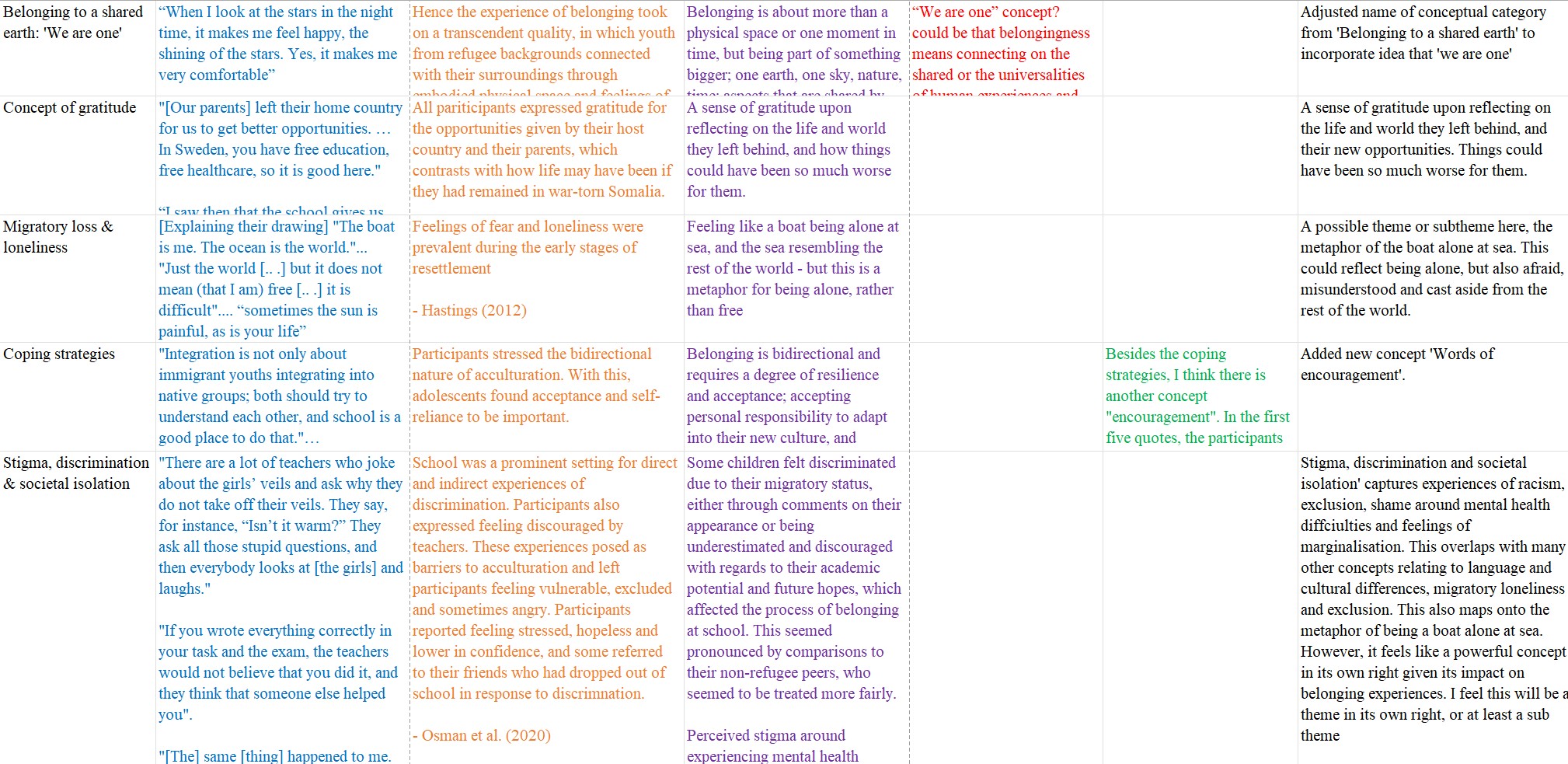


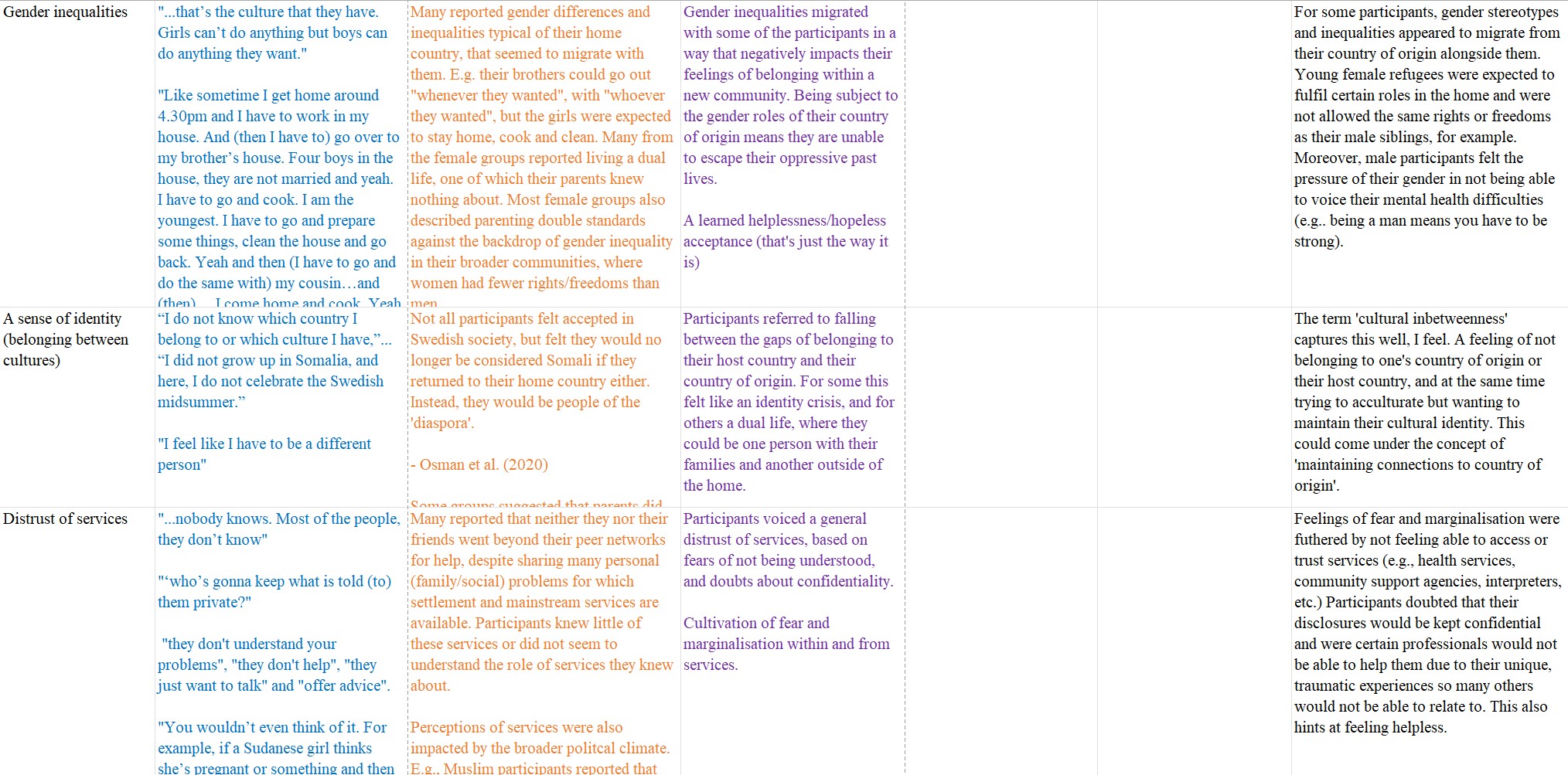


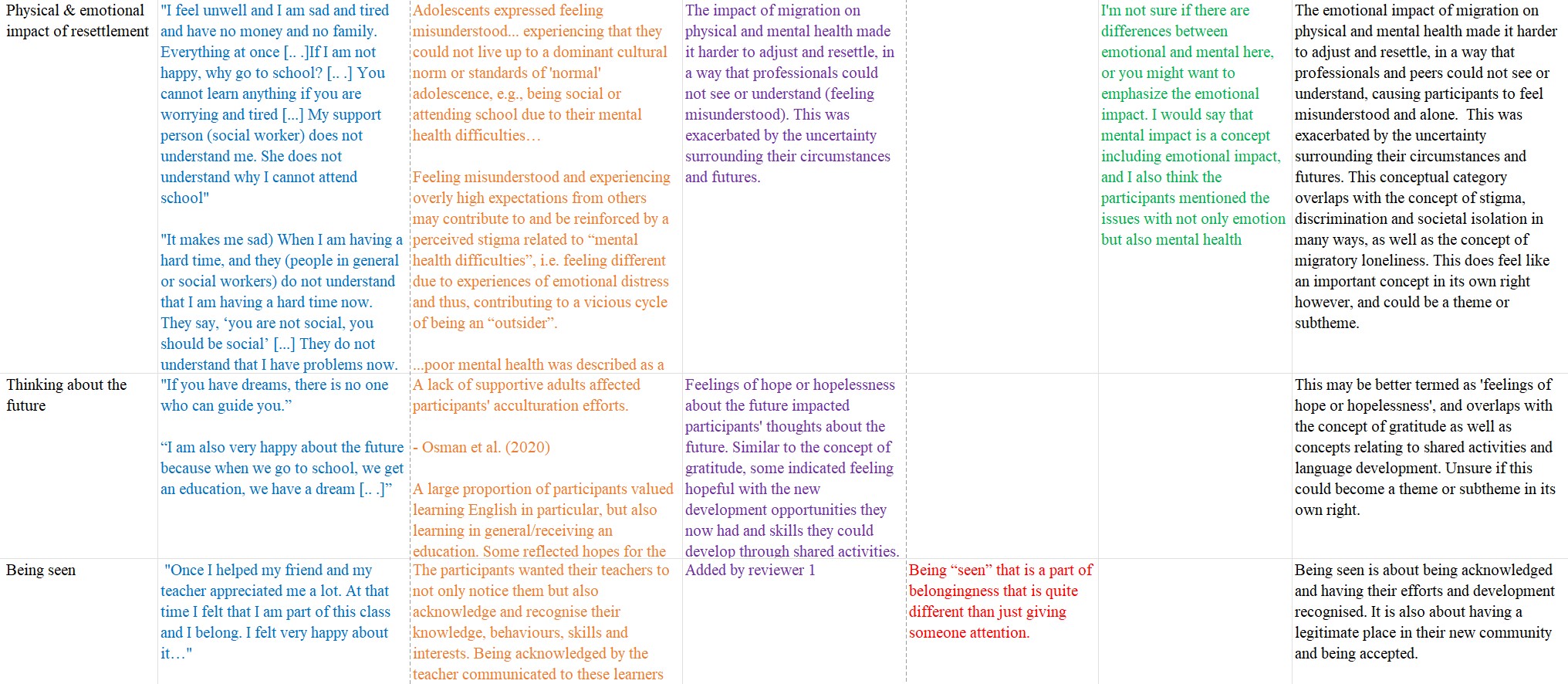


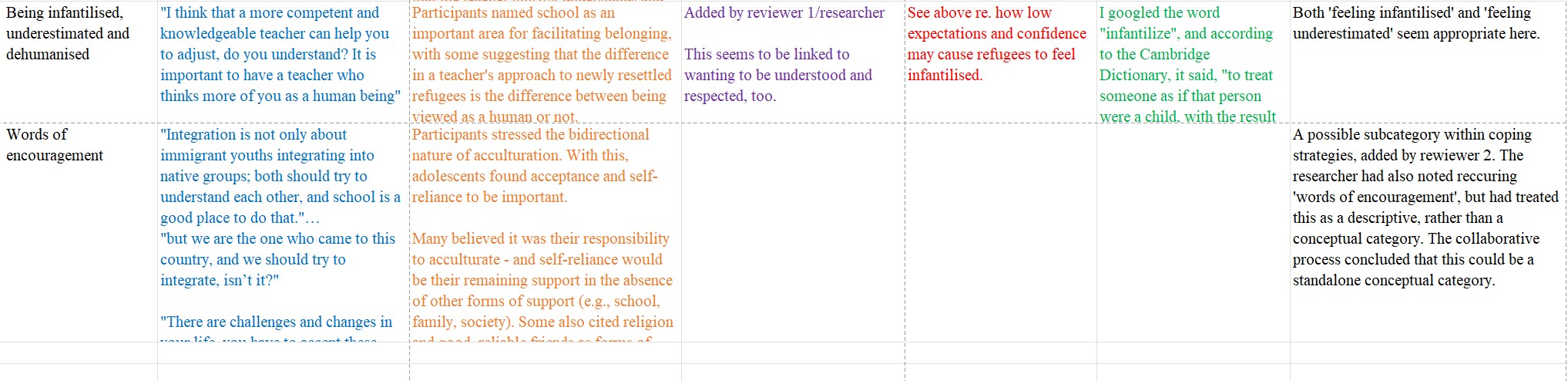


**Supplementary Materials H**

Table H1

*Synthesising Translations*

| **Study**  **Key concepts** | **1** | **2** | **3** | **4** | **5** | **6** | **7** | **8** | **No. of instances** | **Shared patterns of meaning (synthesising translations) and quotes for potential theme names** |
| --- | --- | --- | --- | --- | --- | --- | --- | --- | --- | --- |
| Support & inclusion |  |  |  |  |  |  |  |  | 7 | Feeling included/a felt sense of inclusion because of friends and appreciation from teachers.  Authors interpretation: a sense of emotional stability through inclusion, help and support of friends. |
| Exclusion |  |  |  |  |  |  |  |  | 8 | Feeling unsupported at school. Feeling ignored, invisible and like teachers don’t care, “It seems I am invisible” |
| Seeking protection |  |  |  |  |  |  |  |  | 4 | Friends are protectors/ Friends provide comfort and protection.  Family bonds also offer security. |
| Helping others, feeling needed & appreciated |  |  |  |  |  |  |  |  | 5 | Feeling appreciated for helping others/ Feeling able to contribute to society if accepted… “if society accepts you, you feel included and can contribute to positive things”.  Wanting to help others in same position. |
| Family connectedness |  |  |  |  |  |  |  |  | 4 | Importance of family bonds/membership for creating a sense of security and belonging/togetherness.  "family relationship is the most important thing that is affecting each and every aspect of our life". |
| Severed relationships & disconnection |  |  |  |  |  |  |  |  | 3 | A felt sense of distance, disconnection and yearning through physical separation from family.  Or feeling disconnected from families due to a lack of support and differing attitudes to adjustment. |

| **Study**  **Key concepts** | **1** | **2** | **3** | **4** | **5** | **6** | **7** | **8** | **No. of instances** | **Shared patterns of meaning (synthesising translations) and quotes for potential theme names** |
| --- | --- | --- | --- | --- | --- | --- | --- | --- | --- | --- |
| Shared activities |  |  |  |  |  |  |  |  | 3 | Inclusion and confidence promoted through shared, meaningful activities and group membership. A strategy which “helps you feel happy and able to manage every hardship”, and “learn how to adapt to society”.  Despite the word inclusion this feels separate to the support/inclusion category, and a meaningful translation in its own right (e.g., helpful adjustment strategies). |
| Recommendatio ns for teachers |  |  |  |  |  |  |  |  | 5 | Suggestions for teachers based on exclusion experiences and feeling ignored. Merged into exclusion category and held in mind for later discussion. |
| Concept of language & culture |  |  |  |  |  |  |  |  | 6 | Fitting in means speaking in the same language as my friends who are from the same backgrounds. Importance of shared language, linguistic brokers or opportunities to learn language are important for inclusion (merged into support/inclusion category). |
|  |  |  |  |  |  |  |  |  |  | However, three of these studies (1, 2 & 8) also acknowledged language and cultural barriers; something that can promote feelings of being alone and an outsider. |
| Financial burden |  |  |  |  |  |  |  |  | 2 | Feelings of increased responsibility to look after family during resettlement - and competing choices. For some, this was more about the a need to look after oneself if alone. |
| Maintaining connections to country of origin |  |  |  |  |  |  |  |  | 7 | Maintaining cultural identity, or maintaining attachment/connections to country of origin.  [Belonging means to] “belong to my country"… “It’s something special about my country. I love my country. I think I’ve learnt [many things in] my country, and I have something, some [things I] remember about my country. My friends, my family”. |
| Recreating feelings of family & home |  |  |  |  |  |  |  |  | 2 | “When I am with my friends, I feel like I’m home… They make me feel home, they make me feel better than ever”… “…the school gives us education, gives us whatever we need, so then they are part and parcel of our family” (merge into support/inclusion). |
| Belonging to a shared earth: We are one |  |  |  |  |  |  |  |  | 1 | Belonging as a transcendent quality, or belonging to a larger entity. |

| **Study**  **Key concepts** | **1** | **2** | **3** | **4** | **5** | **6** | **7** | **8** | **No. of instances** | **Shared patterns of meaning (synthesising translations) and quotes for potential theme names** |
| --- | --- | --- | --- | --- | --- | --- | --- | --- | --- | --- |
|  |  |  |  |  |  |  |  |  |  | “It doesn’t matter if you are from Africa, Asia, Europe, South America. Just we are people and we live only in one earth”.  “Being part of a place. The people accept you. It doesn’t matter if you are from outside of Australia and you’re from Africa, Asia, just you are belonging here”… Another strategy? |
| Concept of gratitude |  |  |  |  |  |  |  |  | 1 | A felt sense of gratitude/the ability to dream and look forward... “(our parents) left their home country for us to get better opportunities” |
| Migratory loss & loneliness |  |  |  |  |  |  |  |  | 2 | “Being a foreigner”/”Being a refugee”/”Being an outsider”… “The boat is me, the ocean is the world”.  Feelings of fear, loneliness and being misunderstood…. "I feel like I have to be a different person"…"You always be thinking of going back home cos nobody wants you here”.  "First day I feel like a lone person, like I don’t feel like I belong to this country. When I learn all the laws and about all the people’s behaviours, like I feel now like I belong in this country now."…. “I cannot tell them about my life…”. |
| Coping strategies |  |  |  |  |  |  |  |  | 6 | Belonging as a bidirectional effort/Belonging works both ways/ A sense of responsibility and self-reliance in resettlement (e.g., acceptance, gratitude, engaging in activities, forming relational bonds with others). Helpful strategies. |
|  |  |  |  |  |  |  |  |  |  | On the other hand, some tried to cope through acts of disremembering, or attempts to forget (e.g., self-harm). I.e., unhelpful coping strategies (merge with impact of resettlement on physical and mental health?) |
| Stigma, discrimination & societal isolation |  |  |  |  |  |  |  |  | 4 | Direct and indirect experiences of discrimination/racism and unfairness, in schools and the community. Feeling vulnerable, excluded, angry, stressed and hopeless. Could merge into exclusion category – though this maps into several categories (e..g., being an outsider/foreigner/refugee… feeling alone/misunderstood). |

| **Study**  **Key concepts** | **1** | **2** | **3** | **4** | **5** | **6** | **7** | **8** | **No. of instances** | **Shared patterns of meaning (synthesising translations) and quotes for potential theme names** |
| --- | --- | --- | --- | --- | --- | --- | --- | --- | --- | --- |
|  |  |  |  |  |  |  |  |  |  | This also included feelings of shame and stigma around mental health.  “We will be seen as extremely scum in their mind”… “nobody wants you here”/”nobody wants you around”… “"I don’t wanna be known as the freak from another country that  can’t do anything”… “politicians and the government do not see the human being…” |
| Emotional impact of resettlement on physical and mental health |  |  |  |  |  |  |  |  | 1 | Feeling tired, angry, misunderstood and low in self-esteem/ Feeling unable to communicate with peers… “And that’s when we start thinking that we are just the most useless thing on the planet."  Perceived stigma relating to mental health difficulties. Shame and wanting to conceal problems…coping through “acts of disremembering” or attempting to forget (e.g., through self-harm, drinking, dropping out). Merges with coping strategies (unhelpful). |
| Distrust of services |  |  |  |  |  |  |  |  |  | Negative perceptions of services… /Fear of further marginalisation through accessing services (shaped by cultural expectations and shame around disclosing personal problems outside of the family, and political climate about extremism fears)… “who’s gonna keep  what is told (to) them private?"… "they don't understand your problems", "they don't help"… This may fall under ‘migratory loneliness’/feeling misunderstood. |
| Gender inequalities |  |  |  |  |  |  |  |  | 1 | Double standards against the backdrop of gender inequality in their broader communities. Women have fewer rights than men, who can do what they want – but men also have to be “tough”, they can’t show weakness. The data supporting this may also fit into stigma, discrimination & societal isolation (though more about societal expectations than isolation). This concept in itself may not be translatable. |
| A sense of identity (falling between cultures) |  |  |  |  |  |  |  |  | 3 | Cultural identities changed by migration/ Feeling rejected in host culture/society… “I do not know which country I belong to or which culture I have”… "I feel like I have to be a different person". |

| **Study**  **Key concepts** | **1** | **2** | **3** | **4** | **5** | **6** | **7** | **8** | **No. of instances** | **Shared patterns of meaning (synthesising translations) and quotes for potential theme names** |
| --- | --- | --- | --- | --- | --- | --- | --- | --- | --- | --- |
|  |  |  |  |  |  |  |  |  |  | Conflicting attitudes towards home culture/first language; emotional attachment vs. barrier to adjustment. Merge with maintaining cultural identity. |
| Thinking about the future |  |  |  |  |  |  |  |  | 3 | Hope for the future/ Setting goals for the future/ Embracing prospect of future opportunities – facilitated by opportunities to learn the language and develop new skills. education in host  country. Merge with concept of gratitude and coping strategies (e.g., Feelings of hope, or strategies for moving forward?) |
| Being seen |  |  |  |  |  |  |  |  | 1 | Being acknowledged and recognised by teachers/ Being appreciated for helping others. Merge with support/inclusion. |
| Being infantilised, underestimated  & dehumanised |  |  |  |  |  |  |  |  | 2 | The difference in a teacher's approach to newly resettled refugees is the difference between being viewed as a human or not. |
| Words of encouragement |  |  |  |  |  |  |  |  | 1 | Merged with coping strategies. |

Figure I1

*Researcher Visual ‘Map’ & Textual Line of Argument*

**Supplementary Materials I**


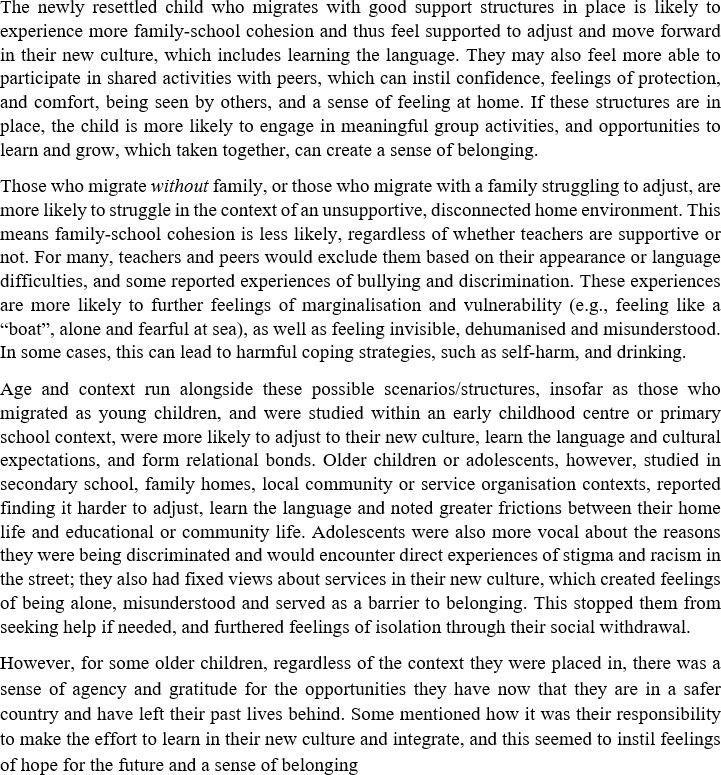
Creating Visual ‘Maps’


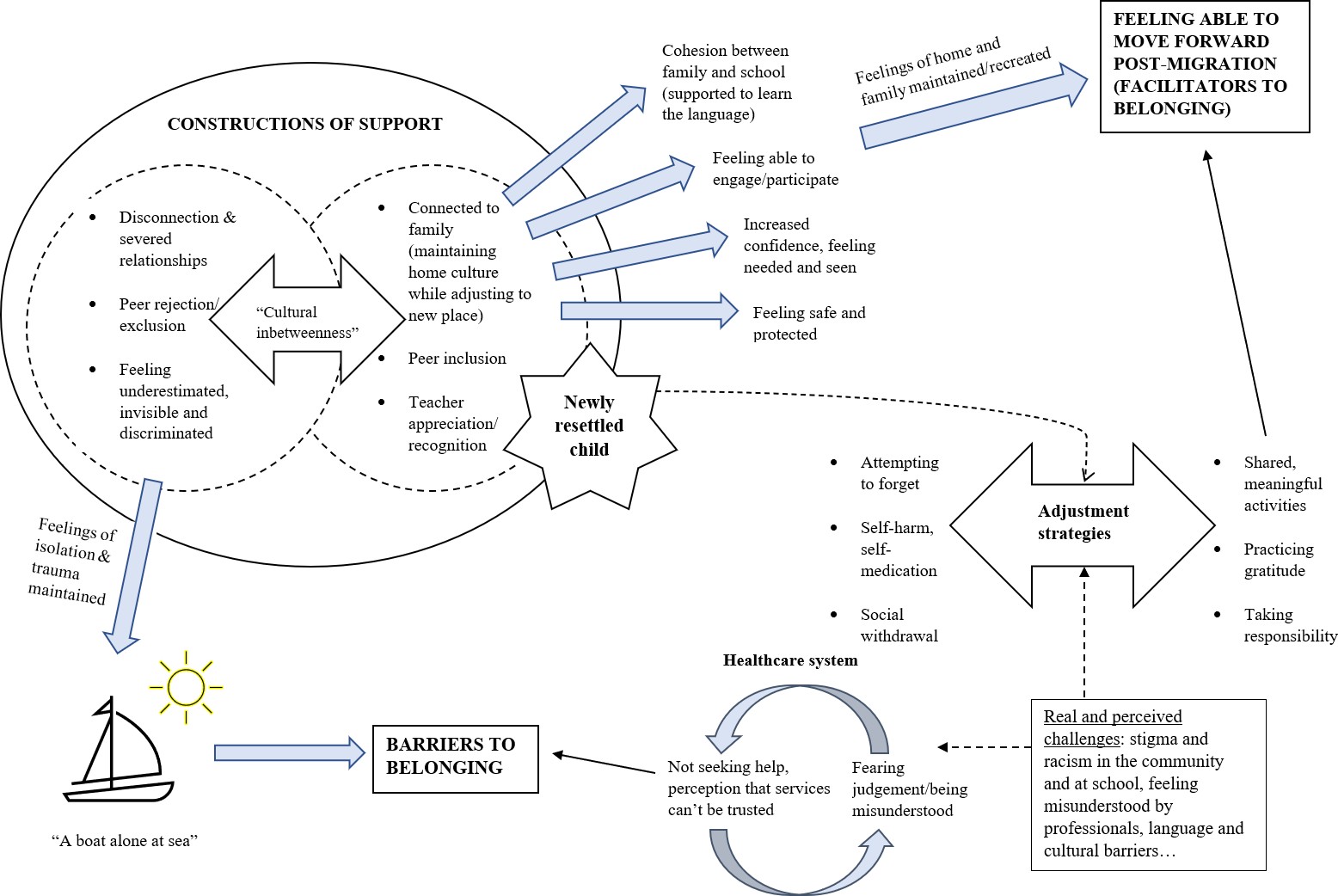


Figure I2

*Research Support Team Visual ‘Map’ & Textual Line of Argument*


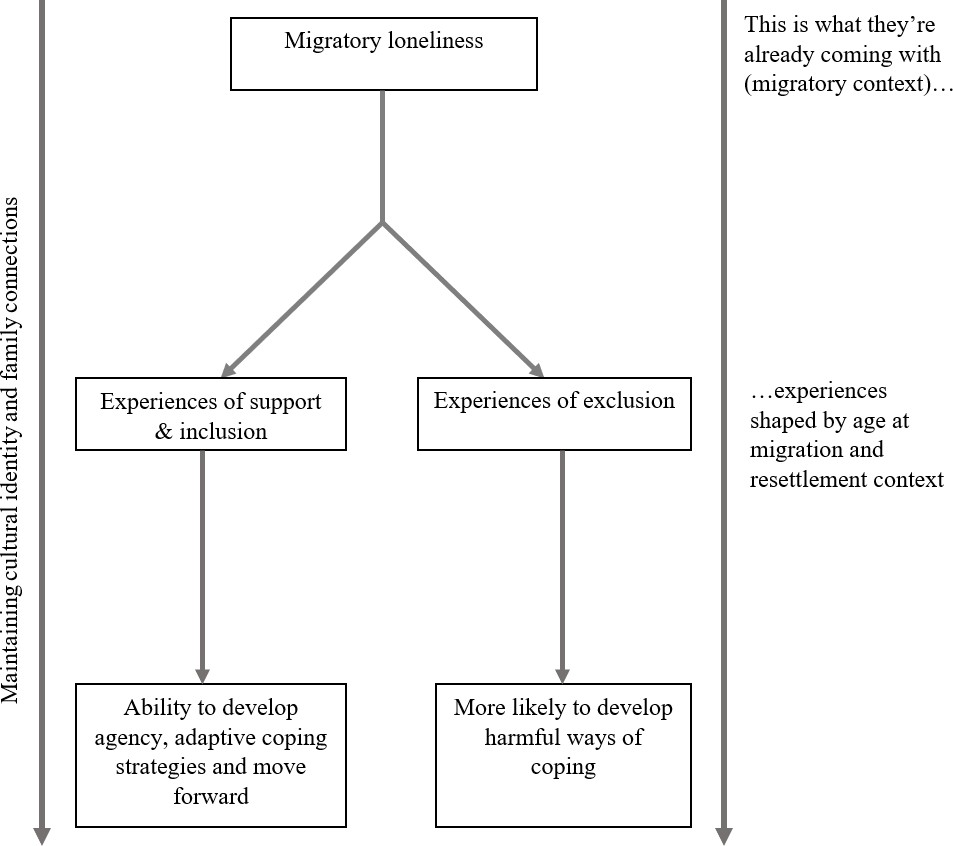
There seems to be a migratory context (migratory loneliness) that young refugees already come with, and then there seems to be a fork in the road where they go on to experience general processes of adolescence.

For some, this may include supportive, inclusive experiences (e.g., friendly peers and teachers who recognise and appreciate them), while others encounter more experiences of exclusion and discrimination (e.g., peer rejection and bullying, teachers who seem to ignore them). These processes seem to be shaped by their age at migration and the context they are resettling in (e.g., younger refugees at primary school find it easier to resettle, learn the language and make friends compared to those resettling during adolescence, at secondary school, the local community or in services).

Running alongside these processes is the idea that some young refugees remain connected to their family and country of origin, and thus maintain their cultural identity, but others feel a sense of falling between cultures and may be disconnected from their family. These experiences then lead to their ability to develop adaptive coping strategies or unhealthy ways of coping.
